# Divergent myeloid and lymphoid immune landscapes in HPV/p16 positive and HPV/p16 negative oropharyngeal squamous cell carcinomas and their lymph node metastases

**DOI:** 10.1186/s10020-026-01481-w

**Published:** 2026-04-30

**Authors:** Raphaela Graessle, Iris Piwonski, Achim Franzen, Heidi Olze, Anja A. Kühl, Michael Hummel, Ulrike Erben, Annekatrin Coordes

**Affiliations:** 1 Department of Otorhinolaryngology, Medical University of Innsbruck, Innsbruck, Austria; 2 Department of Otorhinolaryngology - Head and Neck Surgery, University Hospital Ruppin-Brandenburg, Brandenburg Medical School, Neuruppin, Germany; 3 Department of Pathology, Charité – Universitätsmedizin Berlin, corporate member of Freie Universität Berlin and Humboldt-Universität zu Berlin, Berlin, Germany; 4 Faculty of Health Sciences Brandenburg, Joint Faculty of the University of Potsdam, Brandenburg University of Technology Cottbus-Senftenberg and Brandenburg Medical School, Potsdam, Germany; 5 Department of Otorhinolaryngology, Head and Neck Surgery, Campus Virchow Klinikum and Campus Charité Mitte, Charité – Universitätsmedizin Berlin, corporate member of Freie Universität Berlin and Humboldt-Universität zu Berlin, Berlin, Germany; 6 iPATH. Berlin – Core Unit Immunopathology for Experimental Models, Charité – Universitätsmedizin Berlin, corporate member of Freie Universität Berlin and Humboldt-Universität zu Berlin, Berlin, Germany

**Keywords:** HPV, Oropharyngeal carcinoma, MDSC, Macrophages, Tumor infiltrating lymphocytes

## Abstract

**Background:**

The tumor microenvironment of oropharyngeal squamous cell carcinoma (OPSCC) harbors diverse immune cell populations that influence tumor progression and patient outcome.

**Materials:**

In 102 surgically treated OPSCCs, we determined HPV/p16 status by immunohistochemistry and RNA in situ hybridization. We analyzed mRNA transcripts using the PanCancer IO360 panel by NanoString.

Multiplex immunohistochemistry/immunofluorescence was performed to characterize monocytic (M-MDSC, CD11b⁺CD14⁺HLA-DR^low/−^CD15^−^) and polymorphonuclear myeloid derived suppressor cells (PMN-MDSC, CD11b⁺CD14^−^HLA-DR^low/−^CD15^+^), M1-like (CD68⁺iNOS⁺) and M2-like macrophages (CD68⁺CD206⁺), B cells (CD20⁺), T helper cells (CD3⁺CD4⁺), and cytotoxic T cells (CD3⁺CD8⁺) in the tumor (TC) and stroma compartment (SC) of the primary tumor (PT) and the lymph node metastases (LM).

**Results:**

Transcriptomic profiling revealed higher lymphoid compartment and antigen presentation scores in HPV/p16⁺ OPSCC (*p* = 0.002, *p* = 0.020, respectively), consistent with increased tumor-infiltrating lymphocytes (*p* = 0.025). HPV/p16– OPSCC exhibited higher myeloid compartment scores (*p* < 0.001). M2-like macrophages and M-MDSCs were significantly enriched in PT, while M1-like macrophages and PMN-MDSCs predominated in LM (*p* < 0.001 each). In HPV/p16 + OPSCC, M1-like macrophages, cytotoxic T and B cells were increased (*p* < 0.001, *p* < 0.001, *p* = 0.050, respectively), whereas MDSC frequencies were comparable between both HPV/p16 subgroups. Higher PMN-MDSC infiltration was correlated with poorer overall survival (OS, *p* = 0.050), while increased T helper, cytotoxic T, and B cell infiltration predicted improved OS (*p* = 0.009, *p* < 0.001, *p* = 0.005, respectively).

**Conclusion:**

HPV/p16 + OPSCCs exhibit a lymphoid-dominant, antigen-presenting immune phenotype, whereas HPV/p16– tumors display a myeloid-dominated TME. Despite similar MDSC frequencies, transcriptional and spatial analyses suggest functional divergence of myeloid lineages and local immune differentiation between primary and metastatic sites.

**Supplementary Information:**

The online version contains supplementary material available at 10.1186/s10020-026-01481-w.

## Introduction

Head and neck carcinomas rank among the most prevalent malignancies in Germany. Oropharyngeal cancers constituted 37% of all tumors of the oral cavity and pharynx in 2019. Approximately 84% of carcinomas of the oral cavity and pharynx are squamous cell carcinomas (SCC) (Zentrum für Krebsregisterdaten im Robert Koch-Institut 2022; Barnes et al. 2019). Multiple risk factors contribute to the pathogenesis of SCC in the oropharynx. In addition to the conventional risk factors of tobacco and alcohol consumption, infection with human papillomavirus (HPV) has emerged as an increasingly important etiologic factor, especially in oropharyngeal squamous cell carcinoma (OPSCC) (Graessle et al. 2023; Morse et al. 2007; Schmidt et al. 2004; Taberna et al. 2017). Accordingly, p16 status has been incorporated into the latest Union for International Cancer Control (UICC) tumor classification as a surrogate marker of HPV infection (O'Sullivan 2017). During the past two decades, the 5 year survival rate for OPSCC in Germany has improved only modestly. This trend reflects both decreasing tobacco use among older adults and a rising proportion of HPV associated OPSCC in younger populations (Jansen et al. 2021; Fazel et al. 2020; Statistisches Bundesamt 2021). Clinically, HPV positive OPSCCs are associated with improved prognosis, enhanced responsiveness to chemoradiation, and reduced comorbid burden compared with HPV negative counterparts (Kaplon et al. 2020; Powell et al. 2021; Graessle et al. 2022). Beyond tumor-intrinsic factors, attention has shifted toward the tumor microenvironment (TME), which represents the complex cellular and molecular milieu surrounding malignant cells. The TME comprises immune cell subsets, cancer associated fibroblasts, endothelial cells, pericytes, and other stroma components that dynamically modulate tumor growth, immune evasion, and therapeutic response (de Visser and Joyce 2023). Components of the TME can both promote tumor growth and inhibit its spread, and are increasingly recognized as potential therapeutic targets (Bejarano et al. 2021). Head and neck squamous cell carcinomas (HNSCCs) are typically heavily infiltrated by immune cells, including tumor infiltrating lymphocytes (TILs) and myeloid cell populations, with notable differences between HPV positive and HPV negative tumors (Partlova et al. 2015; Mandal et al. 2016).

Among these, myeloid derived suppressor cells (MDSCs) and tumor associated macrophages (TAMs) play central immunoregulatory roles. MDSCs comprise two main subtypes: monocyte-derived MDSCs (M-MDSCs; CD14^+^CD11b^+^HLA-DR^low/–^CD15^–^) and polymorphonuclear MDSCs (PMN-MDSCs; CD15^+^CD11b^+^CD14^–^) (Veglia et al. 2021; Ruffin et al. 2023; He et al. 2023). Elevated MDSC levels have been observed in HNSCC and are associated with advanced tumor stage and poorer differentiation (Ma et al. 2017). Functionally, MDSCs exert immunosuppressive effects on T cells, dendritic cells, macrophages, and natural killer cells (Gabrilovich et al. 2012).

M-MDSCs suppress antitumor immunity primarily through the production of nitric oxide (NO), the release of immunosuppressive cytokines such as IL-10 and TGFβ, and the expression of regulatory molecules including PDL1. In contrast, PMN-MDSCs rely mainly on reactive oxygen species (ROS), peroxynitrite, arginase 1, and prostaglandin E2 (PGE2) to exert their immunosuppressive effects (Veglia et al. 2021).

TAMs are major contributors to PDL1 expression within the TME (Kurten et al. 2021). They are commonly described as M1-like (antitumoral) and M2-like (protumoral) macrophages, with M2-like TAMs being associated with tumor progression and therapy resistance (Rey-Giraud et al. 2012; Ruffell and Coussens 2015). However, this classification represents an oversimplified model, as TAMs display considerable plasticity and exist along a dynamic functional continuum rather than as two strictly distinct subsets (Chamseddine et al. 2022). Monocytes and M-MDSCs may serve as precursors of M2-like macrophages, thereby linking myeloid recruitment to macrophage polarization within the TME (Kwak et al. 2020).

The present study investigates immune cell components of the OPSCC microenvironment—with a particular focus on MDSCs, TAMs, B cells, CD4⁺ T helper cells, and CD8⁺ cytotoxic T cells. Specifically, our goals were to determine the predominant localization of MDSCs (tumor vs. stroma compartment; primary tumor vs. lymph node metastases), and to evaluate differences in their distribution between HPV positive and HPV negative OPSCC. Additionally, we aimed to assess their association with overall survival (OS).

## Methods

### Patient cohort

This study builds on a previously discussed cohort (Graessle et al. 2025). Archived formalin fixed, paraffin embedded (FFPE) oropharyngeal carcinoma samples were obtained from the Central Biomaterial Bank of Charité – Universitätsmedizin Berlin. A total of 180 patients who underwent resection for operable oropharyngeal carcinoma between 2012 and 2020 at Charité – Universitätsmedizin Berlin were reviewed; 102 cases provided sufficient tissue for the present analyses. All patients underwent standard diagnostic and staging procedures as described in more detail in a previous publication (Graessle et al. 2025). The study was approved by the institutional ethics committee (EA2/005/18).

The following clinicopathological variables were documented: sex (male vs. female), age at initial diagnosis of OPSCC, alcohol abuse (no ethanol consumption vs. ethanol consumption), tobacco exposure (non-smoker vs. former/current smoker), pack years, recurrence (positive vs. negative), grading (G1 vs. G2 vs. G3), lymphatic invasion (L0 vs. L1), venous invasion (V0 vs. V1), perineural invasion (PNI0 vs. PNI1), resection margin (R status; R0 vs. R1), extracapsular spread (ECS; negative vs. positive), T classification (T1-2 vs. T3-4), N classification (positive vs. negative) and stage according to the 8th edition of the TNM Classification of Malignant Tumours published by the UICC (I-II vs. III-IV).

### Determination of HPV/p16 status

Tissue microarrays (TMAs) with two cores (1,5 mm diameter) for each case were constructed from the FFPE surgical specimens of the 102 primary tumors. HPV/p16 status was determined by immunohistochemistry (IHC) and RNA in situ hybridization (RNA-ISH).

IHC was performed according to standard procedures. Hematoxylin was used as counterstaining. For the detection of p16, we used the BenchMark ULTRA Autostainer (Ventana, Tucson, Arizona, USA) and monoclonal rabbit p16 antibodies E6H4 (solution 1:2, Roche/Ventana, Tucson, Arizona, USA). A positive p16 status was defined as a medium-to-strong (2 +/3 +) intensity of nuclear staining with a distribution of ≥ 75% of tumor cells. Cytoplasmic staining was deemed inconsequential.

For RNA-ISH, we used the RNAscope® Probe—HPV-HR18 assay (Advanced Cell Diagnostics, Newark, California, USA), which is able to detect high-risk HPV E6/E7 mRNA of 18 HPV types: 16, 18, 26, 31, 33, 35, 39, 45, 51, 52, 53, 56, 58, 59, 66, 68, 73 and 82.

Both methods produced concordant results, we thus had two groups of HPV/p16 positive und HPV/p16 negative OPSCC patients.

### RNA extraction

RNA was isolated from 10–20 μm FFPE sections using the bead based Maxwell® RSC RNA FFPE Kit (Promega, Madison, WI, USA; Cat. AS1440) according to the manufacturer’s protocol. Tissue sections were scraped, centrifuged, and processed with mineral oil and lysis buffer containing Proteinase K and dye. Following DNase I treatment and incubation, the aqueous phase was transferred to Maxwell® FFPE Cartridges and processed automatically via Maxprep™ Software (Promega Corporation 2022). RNA quantity was measured fluorometrically using the Qubit 4 Fluorometer (Thermo Fisher Scientific, Waltham, MA, USA), and integrity was assessed by fragment analysis.

### Expression analysis with the NanoString nCounter Technology

For mRNA quantification, 300 ng of total RNA per sample was analyzed using the NanoString nCounter Analysis System and the PanCancer IO360 Panel (750 genes + 20 housekeeping genes; NanoString Technologies, Seattle, WA, USA), following the manufacturer’s instructions. Hybridized reporter–capture probe complexes were immobilized and aligned for digital counting across 550 fields of view via the nCounter Digital Analyzer (Geiss et al. 2008).

Raw counts were processed using the nSolver Analysis Software v4.0 and the Advanced Analysis Module (v2.0.134), including the integrated geNorm algorithm for normalization. Outlier detection included principal component analysis, p value distribution, and heat map visualization, leading to exclusion of seven samples. Pathway Score, Differential Expression, Gene Set Analysis, and Cell Type Profiling modules were applied. Analyses focused on three predefined gene sets (lymphoid and myeloid compartments and antigen presentation pathways); the complete list of mRNAs defining each gene set is provided in Supp. Table 1. In Cell Type Profiling, predefined marker mRNAs for macrophages, exhausted CD8⁺ T cells, B cells, CD45⁺ cells, T cells, and CD8⁺ T cells showed significant correlations (see Supp. Table 1), supporting their use in downstream analyses. Cell type scores were generated by the Advanced Analysis module based on predefined gene expression signatures and represent normalized transcriptional enrichment within the bulk RNA dataset. These scores do not correspond to absolute immune cell counts. The composite TIL score is calculated as the arithmetic mean of multiple immune cell-type signature scores and therefore reflects a relative immune context index rather than a hierarchical or summative measure of lymphocyte abundance. For additional statistical testing and visualization, normalized data were exported to RStudio (v2022.12.0 + 353; R v4.2.2).

### Immunohistochemistry/immunofluorescence

To visualize and quantify lymphoid and myeloid populations within tumor and stroma compartments of the primary tumor (PT) and the lymph node metastases (LM), 1–2 mm TMA sections were analyzed at 400 × magnification (10 high-power fields per staining). Cell counts were normalized to total Hematoxylin or DAPI (4′,6-diamidino-2-phenylindole) positive nuclei, and spatial distribution (tumor vs. stroma compartment) was assessed qualitatively. Three multiplex staining protocols were implemented (see Table 1) to identify major immune subsets under multispectral, semi automated imaging conditions. Corresponding antibodies are listed in Table 2. CD33 was included in the staining panel but was not used for final phenotypic classification due to limited co-expression in the multiplex immunofluorescence data.

**Table 1:** Multiplex immunostaining panels for immune cell visualization

Staining		Cell type
1.	CD33, CD11b, CD14, CD15, HLA-DR	M-MDSC, PMN-MDSC
2.	CD20, CD3, CD4, CD8	B cells, T helper cells, cytotoxic T cells
3.	iNOS, CD68, CD206	M1-like macrophages, M2-like macrophages

**Table 2:** Primary antibodies used in this study

Target	Antibody
CD33	clone 6C5/2, Abcam
CD11b	clone EP1345Y, Abcam
CD14	polyclonal rabbit, Sigma
CD15	clone Carb-3, Dako
HLA-DR	clone EPR3692, Abcam
CD20	clone L26, Dako
CD3	polyclonal rabbit, Dako
CD4	clone 4B12, Cell
CD8	clone C8/144B, Dako
iNOS	polyclonal rabbit, Invitrogen
CD68	clone PG-M1, Dako
CD206	clone 5C11, LSBio

Image analysis was performed using inForm software (Akoya Biosciences, version 2.4.8), including spectral unmixing to generate single-marker channels, automated single-cell analysis to segment nuclei and cell boundaries and measure marker expression, and phenotyping to classify individual cells based on their marker profiles. Marker expression was evaluated at the single-cell level, and signals inconsistent with the expected cellular compartment were not considered positive by the algorithm. Cell phenotypes were quantified in R (version 4.0.2) using RStudio (version 1.3.959) with the phenotr and phenotrReports packages (version 0.2.8).

The average cell count per field of view (FOV) was calculated per sample. For immunohistochemical and immunofluorescence analyses, lymphoid populations were defined as B cells, T helper cells, and cytotoxic T cells, whereas M-MDSC, PMN-MDSC, and M1-/M2-like macrophages were classified as myeloid populations based on their hematopoietic lineage.

### Statistical analysis

All statistical analyses were performed using IBM SPSS Statistics v26.0.0.0 for macOS (IBM Corp., Armonk, NY, USA) and R v4.2.2 within RStudio (v2022.12.0 + 353).

Continuous variables with a normal distribution were summarized as mean ± standard deviation, and non-normal variables as median (range).

Differences between unrelated dichotomous variables were tested using the Chi square test, or the Fisher's exact test between groups with normally distributed continuous variables via the t test, and with non normal distributions via the Mann Whitney U test. Related samples were analyzed using the paired t test or the Wilcoxon test, respectively. For NanoString expression data, p values were adjusted for multiple testing using the Benjamini–Hochberg method; other analyses were exploratory.

Kaplan–Meier curves with log rank tests were used to assess the predictive value of clinicopathological variables and immune cell subsets for OS.

Optimal cut offs for continuous immune cell variables were determined using the “Cutoff Finder” tool (Budczies et al. 2012). OS was defined as the interval between the date of diagnosis and death or last follow up. *P* values ≤ 0.05 were considered statistically significant.

## Results

### Patient characteristics

In this study, we examined 102 patients with OPSCC. 46 of these cases were HPV/p16 positive and 56 were HPV/p16 negative. Table 3 summarizes the different patient and tumor characteristics according to HPV/p16 status. The majority of patients were male (71.6%) with a mean age of 62 years (SD = 10). Over three quarters were current or former smokers (77.5%), whereas frequent alcohol consumption was reported in 28.1%. Tumor recurrence occurred in 27.5%. Most tumors were moderately differentiated (G2 = 63.7%) and without evidence of lymphatic (84.3%), venous (95.1%) or perineural (89.2%) invasion. After surgery, 86.3% achieved R0 resection status. While 79.4% were T1–T2 lesions, 74.5% showed nodal involvement; 26 patients had extracapsular spread. No distant metastases were detected. Overall, 77.5% of tumors were diagnosed at UICC stage I–II. Significant differences between HPV/p16 subgroups were observed for sex, alcohol consumption, and UICC stage. HPV/p16 positive patients were more frequently male (82.1% vs. 58.7%; *p* = 0.009), whereas alcohol consumption was higher among HPV/p16 negative patients (44.1% vs. 4.3%; *p* = 0.001). Early tumor stages predominated in HPV/p16 positive cases (94.6% vs. 56.5%; *p* < 0.001).

**Table 3:** Patient and tumor characteristics of the study population according to HPV/p16 status

Variable	Total *N* = 102	HPV/p16 + *N* = 56	HPV/p16 − *N* = 46	*p* value
Sex – n (%)				**0.009**
Female	29 (28.4)	10 (17.9)	19 (41.3)	
Male	73 (71.6)	46 (82.1)	27 (58.7)	
Age at initial diagnosis of OPSCC, years				0.924
Mean (SD)	62 (10)	61 (10)	64 (10)	
Alcohol consumption – n (%)^†^				**0.001**
No	41 (71.9)	22 (95.7)	19 (55.9)	
Yes	16 (28.1)	1 (4.3)	15 (44.1)	
Smoking habits – n (%)^†^				0.338
Former/current smoker	69 (77.5)	33 (73.3)	36 (81.8)	
Non-smoker	20 (22.5)	12 (26.7)	8 (18.2)	
Pack years				0.136
Median (range)	30 (100)	30 (100)	30 (70)	
Recurrence – n (%)				0.133
Positive	28 (27.5)	12 (21.4)	16 (34.8)	
Negative	74 (72.5)	44 (78.6)	30 (65.2)	
**OPSCC characteristics**				
Grading – n (%)				^‡^
G1	1 (1.0)	0 (0)	1 (2.2)	
G2	65 (63.7)	31 (55.4)	34 (73.9)	
G3	36 (35.3)	25 (45.6)	11 (23.9)	
R status – n (%)				0.856
0	88 (86.3)	48 (85.7)	40 (87.0)	
1	14 (13.7)	8 (14.3)	6 (13.0)	
L status – n (%)				0.906
0	86 (84.3)	47 (83.9)	39 (84.8)	
1	16 (15.7)	9 (16.1)	7 (15.2)	
V status – n (%)				0.656^*^
0	97 (95.1)	54 (96.4)	43 (93.5)	
1	5 (4.9)	2 (3.6)	3 (6.5)	
PNI status – n (%)^†^				0.248^*^
0	91 (89.2)	50 (96.2)	41 (89.1)	
1	7 (6.9)	2 (3.8)	5 (10.9)	
T classification – n (%)				0.452
T1-2	81 (79.4)	46 (82.1)	35 (76.1)	
T3-4	21 (20.6)	10 (17.9)	11 (23.9)	
N classification – n (%)				0.051
N > 0	76 (74.5)	46 (82.1)	30 (65.2)	
UICC stage (8th edition) – n (%)				** < 0.001**
I-II	79 (77.5)	53 (94.6)	26 (56.5)	
III-IV	23 (22.2)	3 (5.4)	20 (43.5)	
ECS – n (%)^§^				0.715
Negative	50 (65.8)	31 (67.4)	19 (63.3)	
Positive	26 (34.2)	15 (32.6)	11 (36.7)	

P values in bold indicate statistical significanceat p ≤ 0.05

*N* number, *SD* standard deviation, *OPSCC* oropharyngeal squamous cell carcinoma, *UICC* Union for International Cancer Control, *ECS* extracapsular spread

^†^Alcohol consumption: 45 cases were unknown, 33 of these were HPV/p16 + and 12 were HPV/p16 −; smoking habits: 13 cases were unknown, 11 of these were HPV/p16 + and two were HPV/p16 −; PNI: 4 cases were unknown, all these were HPV/p16 +

^‡^ The requirements to perform a chi-square test were not fulfilled

^*^Fisher's exact test

^§^N = 76 for all cases with lymph node metastasis

### Immune cell composition and pathway profiling by NanoString Analysis

**Fig. 1:**
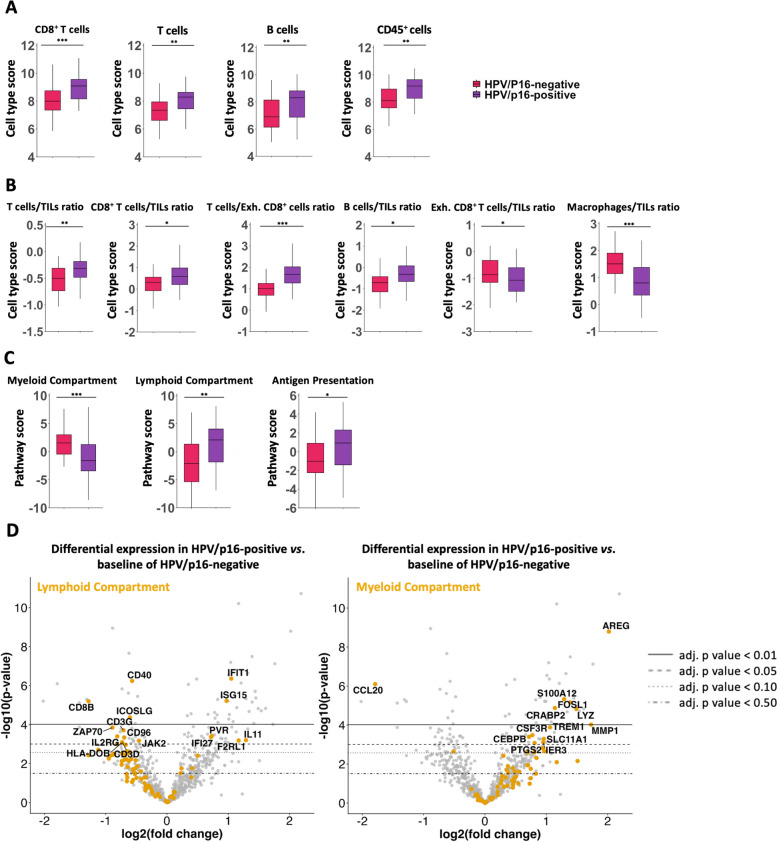
**A** and** B** Gene expression‐based cell types in HPV/p16 positive and HPV/p16 negative OPSCC. **C **Differential expression of pathway genes. **D** Volcano plot depicting significantly increased (right) or decreased (left) expression of immune‐related genes in HPV/p16 negative OPSCC. *p < 0.050, **p < 0.010 and ***p < 0.001

mRNA-based cell type profiling revealed distinct immune landscapes by HPV/p16 status. HPV/p16 positive tumors showed significantly increased infiltration by B cells (*p* = 0.007), T cells (*p* = 0.004), and cytotoxic T cells (*p* < 0.001; Fig. 1A), consistent with increased total CD45 transcript abundance, reflecting overall immune cell load (*p* = 0.006; Fig. 1A).

Comparative ratio analyses revealed that HPV/p16 positive tumors had higher B cell, T cell, and cytotoxic CD8⁺ T cell ratios relative to tumor-infiltrating lymphocytes (TILs), as well as cytotoxic CD8⁺ T cell/exhausted CD8⁺ T cell ratios (*p* = 0.032, 0.002, 0.020, < 0.001, respectively), while exhausted CD8⁺ T cell/TIL and macrophage/TIL ratios were higher in HPV/p16 negative tumors (*p* = 0.032 and < 0.001; Fig. 1B).

Analysis of predefined immune-related pathways confirmed these differences (see Fig. 1C). The myeloid compartment score was significantly higher in HPV/p16 negative OPSCC (p < 0.001), whereas lymphoid and antigen presentation scores were elevated in HPV/p16 positive tumors (*p* = 0.002 and 0.020, respectively). Cytotoxicity pathway scores did not differ between the groups (*p* = 0.334).

Gene set analysis revealed 13 differentially expressed genes (DEGs) in the myeloid, 16 in the lymphoid, and 6 in the antigen presentation groups (Supp. Tables 2–4; Fig. 1D). HPV/p16 negative tumors showed upregulation of most myeloid compartment mRNAs, notably AREG, S100A12, FOSL1, CRABP2, LYZ, and MMP1 (all p < 0.010). Only CCL20 was significantly downregulated (p < 0.001). Conversely, lymphoid compartment and antigen presentation mRNAs were generally downregulated in HPV/p16 negative tumors, contrasting the myeloid profile. Downregulation in the lymphoid compartment was driven mainly by CD40, CD8B, and ICOSLG (all p < 0.010), while HLA-DQA2, CD8B, and HLA-DOB were downregulated in antigen presentation pathways (all p < 0.050).

### HPV/p16 positive vs. HPV/p16 negative OPSCC

**Fig. 2:**
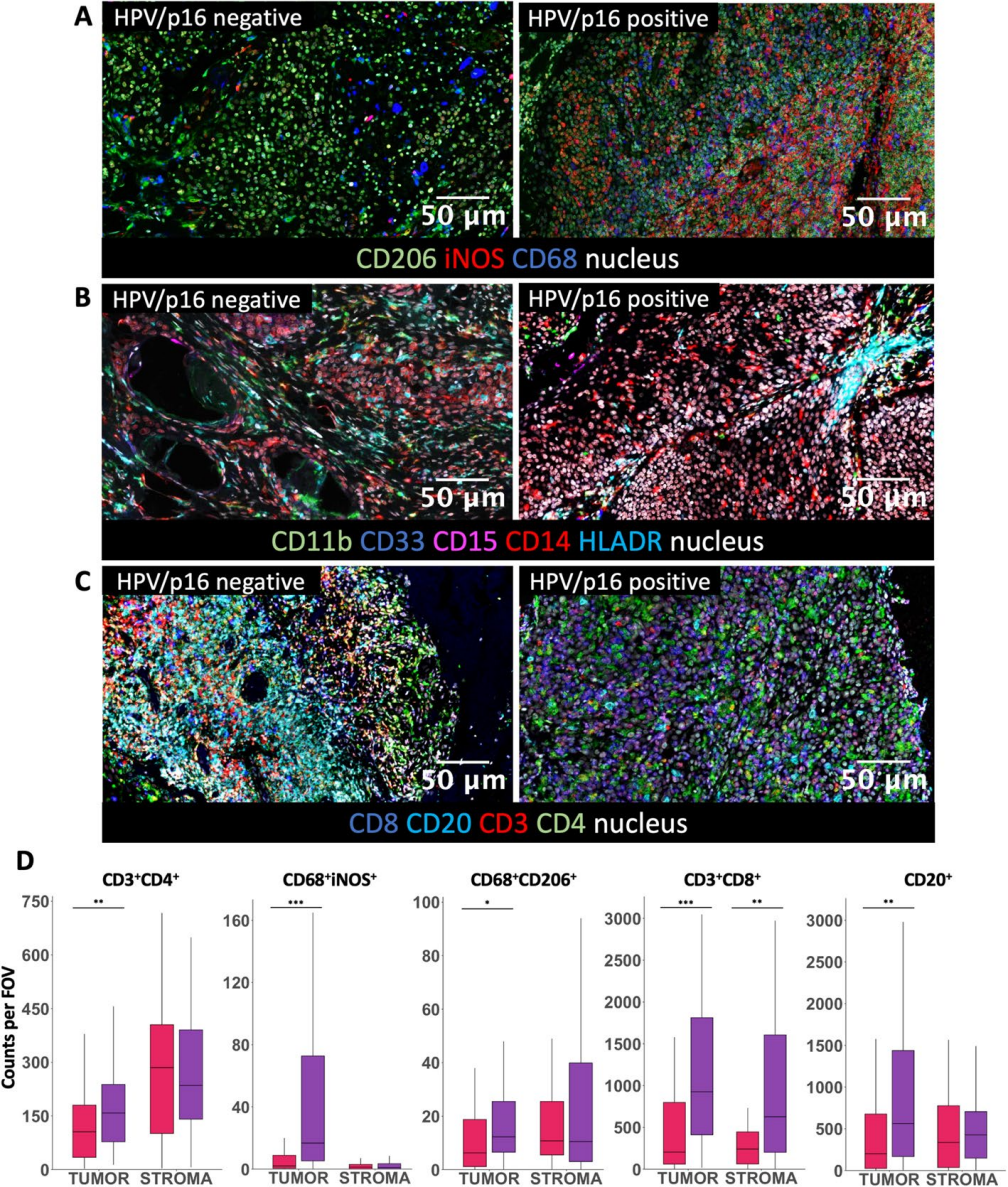
**A**-**C** Representative composite images of multiplex staining showing immune cell markers in HPV/p16 positive and HPV/p16 negative OPSCC. **D** Immune cell populations infiltrating the stroma compartment and tumor compartment of HPV/p16 positive (purple) and HPV/p16 negative (pink) tumors. Counts per FOV represent the average number of cells per field of view per sample. * *p* < 0.050, ** *p* < 0.010 and *** *p* < 0.001

Multiplex immunofluorescence revealed higher densities of CD68⁺iNOS⁺ (M1-like macrophages; median 18.5 vs. 3 cells/FOV, *p* < 0.001), CD3⁺CD8⁺ (cytotoxic T cells; 1689 vs. 450.5 cells/FOV, p < 0.001), and CD20⁺ (B cells; 1104 vs. 675 cells/FOV, *p* = 0.050) in HPV/p16 positive compared with HPV/p16 negative OPSCC (Fig. 2A and C, Supp. Figure 1). In contrast, CD68⁺CD206⁺ (M2-like macrophages), CD11b⁺CD14⁺HLA-DR^low/−^CD15^−^ (M-MDSCs), CD11b⁺CD14^−^HLA-DR^low/−^CD15^+^ (PMN-MDSCs), and CD3⁺CD4⁺ (T helper cells) did not differ significantly (*p* = 0.205, *p* = 0.270, *p* = 0.235, respectively, Figs. 2A-C, Supp. Figures 1–2).

Stratification by tissue compartment revealed significantly higher densities of M1-like macrophages (16.75 vs. 2 cells/FOV, *p* < 0.001), cytotoxic T cells (926.25 vs. 204.5 cells/FOV, *p* < 0.001), and B cells (563.5 vs. 201 cells/FOV, *p* = 0.014) within the tumor compartment of HPV/p16 positive compared with HPV/p16 negative OPSCC, consistent with the overall analysis (Fig. 2D). Additional increases in M2-like macrophages (12.25 vs. 6.25 cells/FOV, *p* = 0.029) and T helper cells (158.00 vs. 105.50 cells/FOV, *p* = 0.004) were observed specifically within the tumor compartment of HPV/p16 positive tumors (Fig. 2D). Stroma compartment differences were limited, with higher cytotoxic T cell infiltration in HPV/p16 positive tissue (627.50 vs. 241.50 cells/FOV, *p* = 0.003, Fig. 2D), while no differences were observed for M-MDSCs or PMN-MDSCs in either tumor or stroma compartments.

### Primary tumor vs. lymph node metastasis

**Fig. 3:**
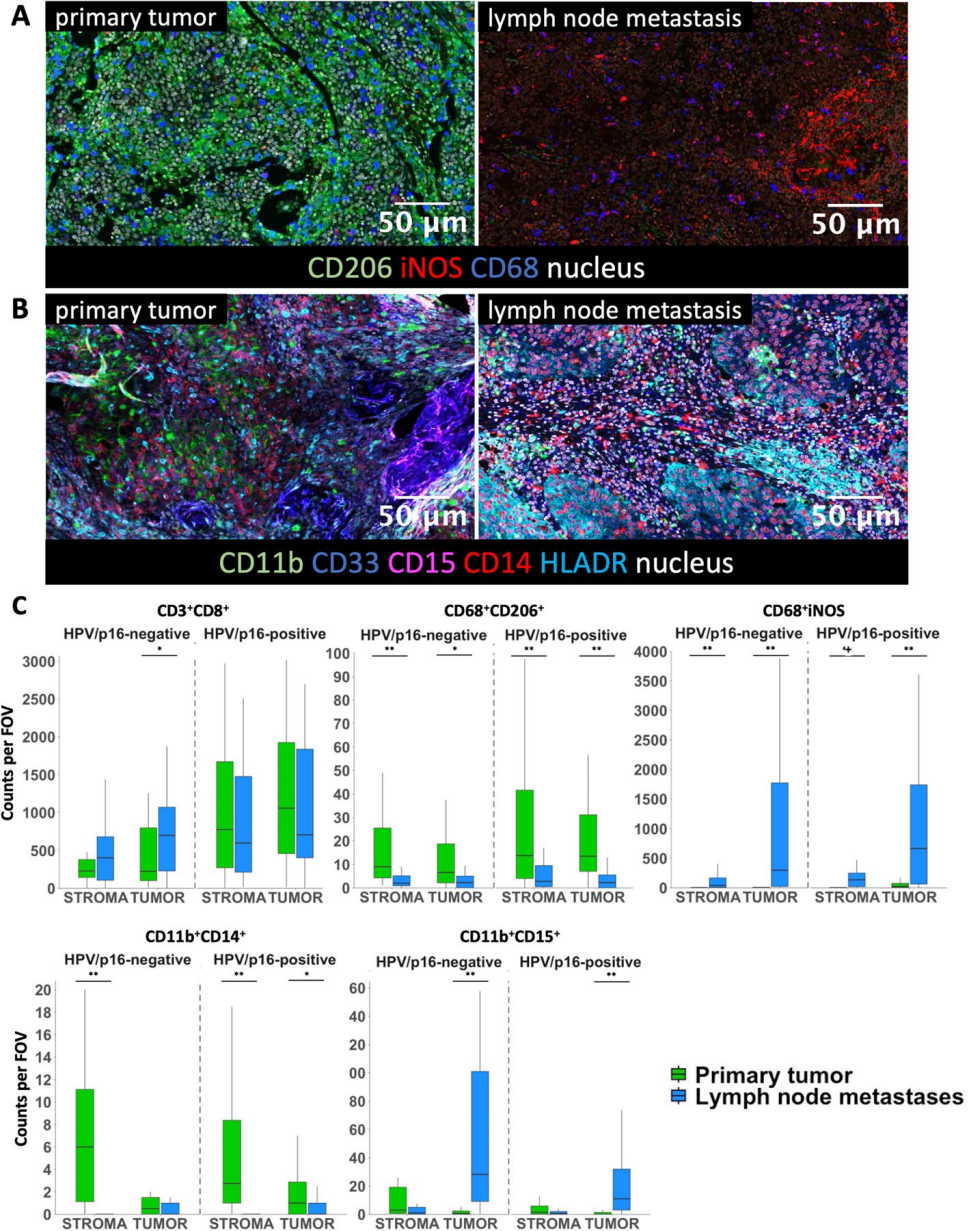
**A** and **B** Representative composite images of multiplex staining showing immune cell markers in the primary tumor and the lymph node metastases. **C** Immune cell populations infiltrating the primary tumor (green) and the lymph node metastases (blue) in the stroma compartment and tumor compartment of HPV/p16 positive and HPV/p16 negative OPSCC. Counts per FOV represent the average number of cells per field of view per sample. * *p* < 0.050 and ** *p* < 0.001. For simplicity, CD11b⁺CD15⁺ and CD11b⁺CD14⁺ are abbreviated; full phenotypes are CD11b⁺CD14⁻HLA-DR^low/−^CD15⁺ and CD11b⁺CD14⁺HLA-DR^low/−^CD15⁻, respectively

Immune composition differed between PT and corresponding LM (Fig. 3). Independent of HPV/p16 status, M2-like macrophages and M-MDSCs were enriched in PT, whereas M1-like macrophages and PMN-MDSCs predominated in LM (M2-like macrophages: HPV + *p* < 0.001, HPV − *p* = 0.003; M-MDSCs, M1-like macrophages and PMN-MDSCs: both *p* < 0.001; Figs. 3A and B, Supp. Figure 3). T helper cells and B cells did not differ between PT and LM, while cytotoxic T cells were increased in LM only in HPV/p16 negative cases (*p* = 0.016).

Compartment-specific analyses further refined these patterns (Fig. 3C). In HPV/p16 positive cases, M2-like macrophages and M-MDSCs were enriched in both tumor and stroma compartments of PT, whereas M1-like macrophages were more frequent in both compartments of LM (all *p* < 0.001; M-MDSCs in the tumor compartment *p* = 0.025). PMN-MDSCs were increased in the tumor compartment of LM (*p* < 0.001) but did not differ in the stroma compartment (*p* = 0.166). In HPV/p16 negative cases, M2-like macrophages predominated in the tumor and stroma compartments of PT (tumor: *p* = 0.036; stroma: *p* = 0.001), while M1-like macrophages were enriched in both compartments of LM (both *p* < 0.001). PMN-MDSCs were increased in the tumor compartment of LM (*p* < 0.001) with no stromal difference (*p* = 0.075). M-MDSCs were comparable between PT and LM in the tumor compartment (*p* = 0.305) but enriched in the stroma compartment of PT (*p* < 0.001), and cytotoxic T cells were increased in the tumor compartment of LM only (*p* = 0.010; stroma *p* = 0.145). Median cell counts per FOV for all subgroup comparisons are provided in Supp. Table S5.

### Tumor vs. stroma compartment

**Fig. 4:**
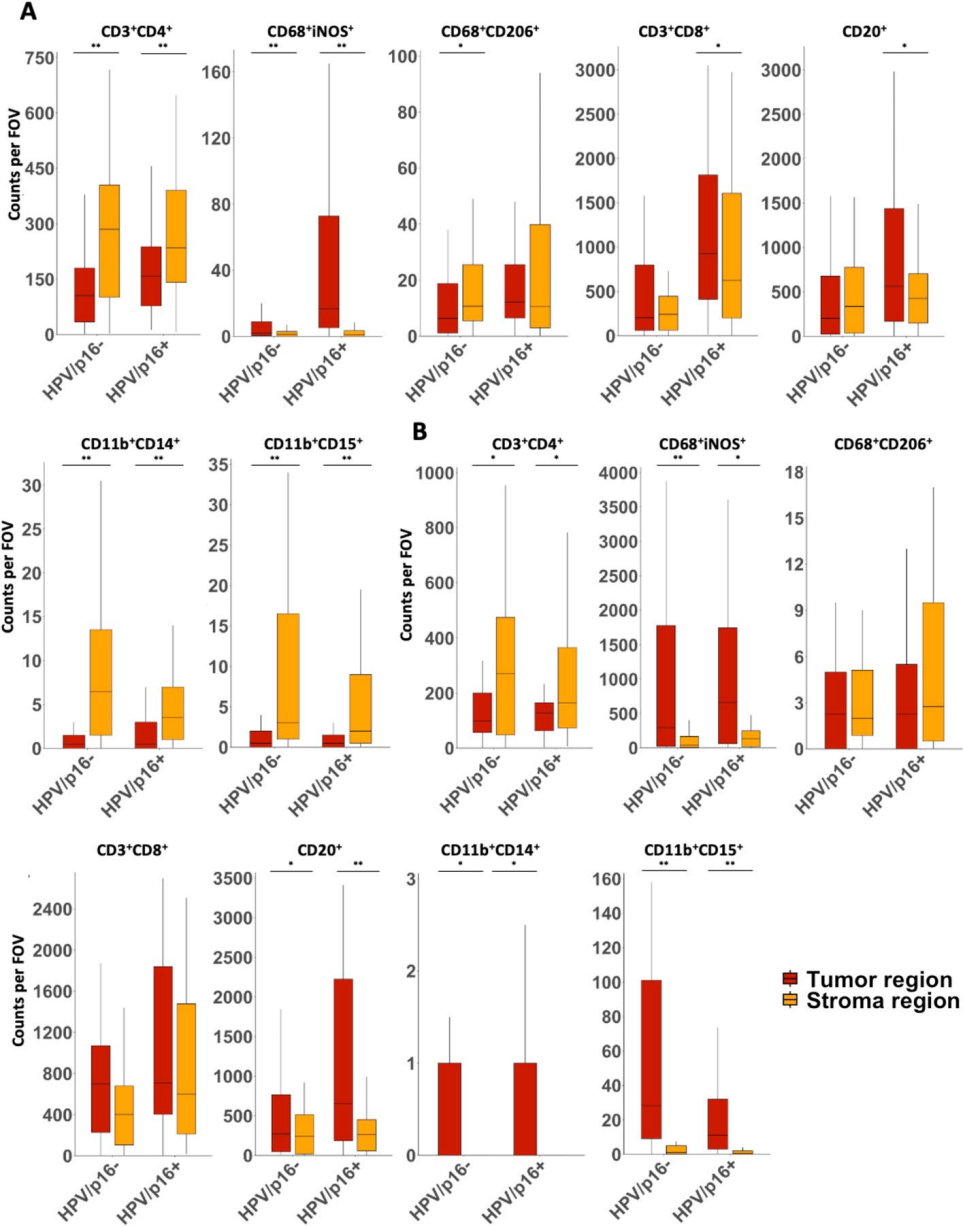
**A** Immune cell populations in the primary tumor that infiltrate the tumor compartment (red) and the stroma compartment (orange) in both HPV/p16 positive and HPV/p16 negative OPSCC. **B** Immune cell populations in lymph node metastases that infiltrate the tumor compartment (red) and the stroma compartment (orange) in both HPV/p16 positive and HPV/p16 negative OPSCC. Counts per FOV represent the average number of cells per field of view per sample. * *p* < 0.050 and ** *p* < 0.001. Abbreviated marker combinations are shown in A and B: CD11b⁺CD15⁺ and CD11b⁺CD14⁺ correspond to CD11b⁺CD14⁻HLA-DR^low/−^CD15⁺ and CD11b⁺CD14⁺HLA-DR^low/−^CD15⁻, respectively

Within primary tumors, tumor compartments were enriched in M1-like macrophages, whereas stroma compartments showed higher infiltration by T helper cells and M- and PMN-MDSCs across both HPV/p16 subgroups (all *p* < 0.001; Fig. 4A). In addition, cytotoxic T cells and B cells preferentially localized to the tumor compartment in HPV/p16 positive—but not HPV/p16 negative—cases (cytotoxic T cells: *p* = 0.014 vs. 0.343; B cells: *p* = 0.032 vs. 0.460), while M2-like macrophages were enriched in the stroma compartment only in HPV/p16 negative tumors (*p* = 0.004 vs. 0.857; Fig. 4A).

In LM, M1-like macrophages and B cells were enriched in tumor compartments, whereas T helper cells predominated in stroma compartments, irrespective of HPV/p16 status (M1-like macrophages: HPV + *p* = 0.002, HPV − *p* < 0.001; B cells: HPV + *p* < 0.001, HPV − *p* = 0.022; T helper cells: HPV + *p* = 0.004, HPV − *p* = 0.002; Fig. 4B). In contrast to primary tumors, M- and PMN-MDSCs were more abundant in tumor than stroma compartments in lymph node metastases (M-MDSCs: HPV + *p* = 0.006, HPV − *p* = 0.019; PMN-MDSCs: both *p* < 0.001), while cytotoxic T cells and M2-like macrophages showed no compartment-specific differences (Fig. 4B). Median cell counts per FOV with ranges and p values are listed in Supp. Tables 6 and 7.

### Overall survival and immune correlates

**Fig. 5:**
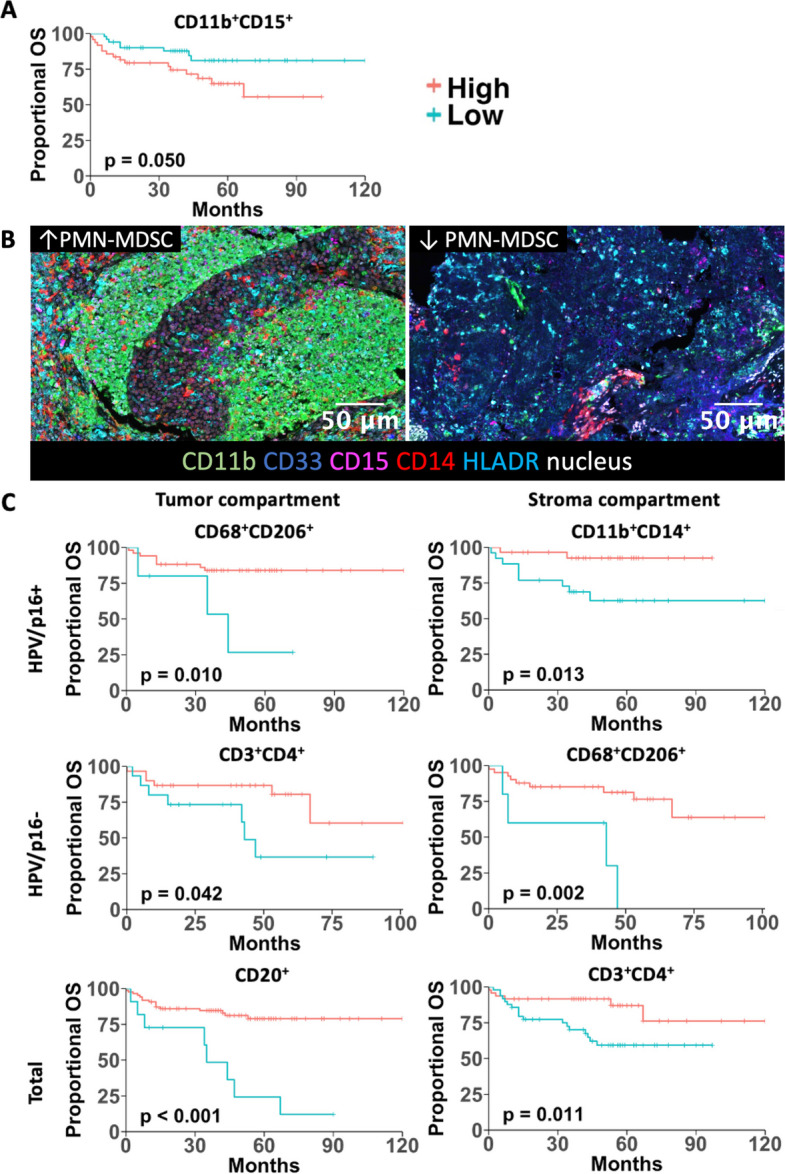
**A** Non compartment-specific Kaplan–Meier curve for overall survival according to PMN-MDSC infiltration. **B** Representative composite images of multiplex staining illustrating tumors with high or low PMN-MDSC infiltration. PMN-MDSCs were defined as CD11b⁺CD14⁻HLA-DR^low/−^CD15⁺ cells. The left panel shows a representative tumor with high PMN-MDSC infiltration (448.00 cells per FOV), whereas the right panel shows a tumor with low PMN-MDSC infiltration (0.50 cells per FOV). The cut-off for high PMN-MDSC infiltration was 3.25 cells per FOV and was applied in the Kaplan–Meier analyses. **C** Compartment-specific Kaplan–Meier curves for overall survival according to immune cell composition, stratified by HPV/p16 status. For simplicity, CD11b⁺CD15⁺ and CD11b⁺CD14⁺ are abbreviated; full phenotypes are CD11b⁺CD14⁻HLA-DR^low/−^CD15⁺ and CD11b⁺CD14⁺HLA-DR^low/−^CD15⁻, respectively

The mean OS was 92 months (95% confidence interval 82.0–102.0), with 1-, 3-, and 5-year OS rates of 89.2%, 81.7%, and 73.8%. Higher infiltration of cytotoxic T cells, T helper cells, and B cells was associated with improved OS in the HPV/p16-total group and in HPV/p16 negative tumors (Supp. Figure 4 A and C, Supp. Tables 8). PMN-MDSCs were linked to poorer OS only in the HPV/p16-total group (Fig. 5A and B, Supp. Figure 5). M2-like macrophages and M-MDSCs improved OS in the HPV/p16-total group and in HPV/p16 positive tumors, whereas M1-like macrophages showed no association (Supp. Figure 4 A and B, Supp. Tables 8).

Compartment-specific analyses largely mirrored these patterns. In the tumor compartment, cytotoxic T cells (HPV total *p* < 0.001, HPV − *p* = 0.004) and T helper cells (HPV total *p* < 0.001, HPV − *p* = 0.042) were prognostic in HPV/p16-total and HPV/p16 negative groups. M2-like macrophages improved OS in HPV/p16-total and HPV/p16 positive tumors (*p* = 0.004 and 0.010), and B cells were prognostic irrespective of HPV/p16 status (HPV total < 0.001, HPV + *p* = 0.006, HPV − *p* = 0.014, Fig. 5C, Supp. Tables 8).

In the stroma compartment, cytotoxic T cells, M2-like macrophages, T helper cells, and M-MDSCs were associated with improved OS in most HPV/p16 subgroups (cytotoxic T cells: HPV total *p* < 0.001, HPV − *p* = 0.004; M2-like: HPV total *p* < 0.001, HPV − *p* = 0.002, HPV + *p* = 0.009; T helper cells: HPV total *p* = 0.011, HPV − *p* = 0.048, HPV + *p* = 0.049; M-MDSCs: HPV total *p* = 0.007, HPV + *p* = 0.013, Fig. 5C, Supp. Tables 8). PMN-MDSCs and M1-like macrophages showed no consistent association with OS in compartment-specific analyses.

## Discussion

This study aimed to characterize the spatial distribution of MDSCs in OPSCC, comparing tumor and stroma compartments and primary tumors versus lymph node metastases, and to examine their relationship with HPV/p16 status.

Analysis of 102 patients revealed that M2-like macrophages and M-MDSCs were enriched in primary tumors, whereas M1-like macrophages and PMN-MDSCs predominated in lymph node metastases. Transcriptomic profiling confirmed distinct immune signatures: HPV/p16 positive tumors exhibited a lymphoid dominant, immunologically activated phenotype, while HPV/p16 negative OPSCCs displayed a myeloid dominated, immunosuppressive microenvironment with enhanced macrophage signatures—findings consistent with previous reports (Oguejiofor et al. 2015; Gameiro et al. 2022; Kansy et al. 2023; Matlung et al. 2016; Wang et al. 2023).

Despite the fact that solid tumors are known to consist of two distinct but interdependent compartments, the tumor and the stroma compartment, separate analyses of immune cell infiltration are rarely performed (Mueller and Fusenig 2004). Although there are studies that deal with the TME of HNSCCs, some of which even include separate analysis of tumor and stroma compartments, to our knowledge there are no studies that focus on the immune cells examined in our work with a focus on the differences between primary tumors and lymph node metastases (Kim et al. 2024; Sadeghirad et al. 2022; Puram et al. 2017).

It has already been shown in prostate cancer that PMN-MDSCs occur more frequently in the stroma than in the tumor compartment and that the stroma compartment of lymph node metastases are more infiltrated than the stroma compartments of primary tumors (Wen et al. 2020). Our work largely supports this finding for OPSCC as well. As with prostate cancer, we found PMN-MDSCs mainly in the stroma compartment. However, we also observed an increased accumulation of M-MDSCs in the stroma compartment. We also corroborate the finding that lymph node metastases are to a larger extend infiltrated by PMN-MDSCs compared to primary tumors in OPSCCs. However, we cannot fully agree that this result applies to the stroma compartment. In our study population, PMN-MDSCs showed significantly higher infiltration in lymph node metastases in the tumor compartment and in the entire tissue (tumor and stroma compartment combined). Unlike in prostate cancer, the level of infiltration by PMN-MDSCs in the respective stroma compartments did not differ significantly when comparing lymph node metastases to the primary tumor (Wen et al. 2020). One possible reason for this difference, apart from the fact that a different tumor entity was examined, could be the size of the comparison group. Even though the number of patients examined was approximately the same, we analyzed HPV negative and HPV positive OPSCC separately in our study, which reduced our group size. In addition, we found an accumulation of MDSCs in the stroma compartment only in the primary tumor. In the lymph node metastases, MDSCs occurred more frequently in the tumor compartment. In this respect, our results also differed from those found for prostate cancer.

Experimental data by Lahmar et al. indicate that M-MDSCs are abundant in tumor draining lymph nodes in non-metastatic settings (Lahmar et al. 2021). In our human cohort, M-MDSC levels were higher in primary tumors than in metastases, possibly reflecting differentiation of M-MDSCs into TAMs under local maturation signals (Corzo et al. 2010; Bayik et al. 2018).

We showed that M2-like macrophages were predominately found in the primary tumor where hypoxia and tumor derived cytokines such as CSF-1, IL-10, and TGF-β favor M2 polarization and promote angiogenesis and tumor invasion (Zhang et al. 2017; Noy and Pollard 2014). In contrast, the lymph node microenvironment, rich in IFN-γ and other pro inflammatory mediators, may restrict M2-like retention or drive their repolarization toward an M1-like phenotype, explaining the higher M1-like macrophage abundance in metastases (Mhaidly et al. 2023; Gocher et al. 2022; Furgiuele et al. 2022; Chen et al. 2023; Chaurasia et al. 2025).

Previous evidence suggests that macrophage density, especially of M2-like phenotype, is greater in stroma than tumor compartments across several solid cancers (Vayrynen et al. 2021; Jackute et al. 2018). In line with this, M2-like macrophages in our study concentrated in stroma compartments of HPV/p16 negative OPSCC, while M1-like macrophages were largely confined to tumor compartments irrespective of HPV status. These patterns are consistent with M1-like driven antitumor immunity and a stromal accumulation of M2-like cells promoting angiogenesis and extracellular matrix remodeling (Rőszer 2015; Mantovani et al. 2002).

A limitation of our study is that mRNA-based profiling was restricted to primary tumors, precluding compartment- or metastasis-specific transcriptomic comparisons. Additionally, identification of PMN-MDSCs in tissue sections is limited by their phenotypic similarity to conventional neutrophils. Accordingly, PMN-MDSCs are increasingly considered pathologically activated neutrophils with immunosuppressive functions (Zhou et al. 2018; Raskov et al. 2022).

In this study, we used the commonly applied marker combination CD11b⁺CD14⁻HLA-DR^low/−^CD15⁺ to define human PMN-MDSCs (Bronte et al. 2016). However, these markers cannot fully discriminate neutrophils from PMN-MDSCs. Functional assays demonstrating suppression of T-cell responses remain the gold standard for confirming MDSC identity but are not feasible in retrospective tissue-based analyses (Bronte et al. 2016). Future projects integrating spatial transcriptomics or single cell RNA sequencing together with functional validation will be required to overcome these limitations and to better resolve cell–cell interactions and signaling dynamics across metastatic niches.

Importantly, our findings corroborate prior work demonstrating enhanced infiltration of cytotoxic T cells and B cells in HPV positive OPSCC and their positive association with OS (Oguejiofor et al. 2015; Wallis et al. 2015; Hladíková et al. 2019; Young et al. 2024; Chen et al. 2018). mRNA analysis corroborated the multiplex staining results, revealing an increased lymphoid and antigen-presenting signature in HPV positive tumors and underscoring a more immunologically active TME in HPV positive OPSCC. Consistent with prior reports of an increased M1/M2 macrophage ratio, we observed increased M1-like macrophage infiltration in HPV/p16 positive tumors, while M2-like infiltration remained unchanged (Chen et al. 2018). Nonetheless, literature remains inconsistent regarding compartment specific distributions (Snietura et al. 2020; Seminerio et al. 2018). Snietura et al. observed enhanced M1/M2 macrophage infiltration in the HPV positive stroma compartment, whereas we detected higher M1/M2 densities within the tumor compartment (Snietura et al. 2020). No significant differences in MDSC infiltration were observed between HPV groups, in line with some but not all reports (Kansy et al. 2023; Yang et al. 2023). Differences in the TME are likely driven by functional activity and differentiation status rather than absolute MDSC numbers. This is supported by our mRNA analyses showing an increased myeloid signature in HPV negative tumors.

In our cohort, strong PMN-MDSC infiltration was associated with poorer OS, consistent with previous reports (Lang et al. 2018).

Conversely, strong T helper, cytotoxic T cell, and B cell infiltration was associated with prolonged survival, supporting the concept of immune activation as a favorable prognostic marker in OPSCC (Oguejiofor et al. 2015; Hladíková et al. 2019).

In contrast to several previous studies reporting M2-like macrophages as negative prognostic markers in HNSCC, we observed an association between higher CD68^+^CD206^+^ macrophage density and improved OS (Bisheshar et al. 2022). Several methodological factors may contribute to this discrepancy. First, optimal cut-offs were determined using the Cutoff Finder algorithm, which resulted in unequal group sizes and may therefore reflect cohort-specific distributions (Budczies et al. 2012). Second, macrophage quantification in the present study was performed on tissue microarrays, which may introduce sampling bias due to the spatial heterogeneity of immune cell infiltration within tumors. Beyond methodological factors, our findings also suggest a biological interpretation: M2-like macrophages may serve as markers of an immunologically active tumor microenvironment rather than direct drivers of survival. This interpretation is supported by the consistent association of higher M2 density with better survival across tumor, stroma, and combined compartments.

## Conclusion

In summary, this study delineates distinct spatial and molecular immune landscapes in HPV/p16 positive and HPV/p16 negative OPSCC:

1. Spatial distribution: M2-like macrophages and M-MDSCs predominated in primary tumors, whereas M1-like macrophages and PMN-MDSCs were enriched in lymph node metastases.
2. Immune composition: HPV/p16 negative OPSCC displayed higher myeloid compartment scores, reflecting a more immunosuppressive TME, whereas HPV/p16 positive tumors showed enhanced lymphoid and antigen presentation signatures consistent with active immune surveillance.
3. Prognostic implications: High PMN-MDSC infiltration was associated with reduced OS, while robust T and B cell infiltration correlated with improved outcomes.

### These findings highlight

The dual roles of myeloid and lymphoid immunity in OPSCC progression and underscore the need for therapeutic approaches that target myeloid immunosuppression while preserving lymphoid activation. Future research should investigate cell–cell interactions, signaling pathways, and differentiation mechanisms within the OPSCC microenvironment to uncover novel immune modulating strategies.

## Supplementary Information

Additional file 1: Supp. Figure 1 Description of data: A. and C. Low-magnification composite images of multiplex immunofluorescence staining showing immune cell markers in HPV/p16 positive and HPV/p16 negative OPSCC, with corresponding zoom-in insets. In A, blue arrows indicate CD68⁺CD206⁺ cells, and orange arrows CD68⁺iNOS⁺ cells. In C, blue arrows indicate CD3⁺CD8⁺ cells, yellow arrows CD20⁺ cells, and green arrows CD3⁺CD4⁺ cells. B. and D. Corresponding phenotype maps of the same fields of view illustrating the automated classification of immune cell populations.

Additional file 2: Supp. Figure 2 Description of data: A. Low-magnification composite images of multiplex immunofluorescence staining showing immune cell markers in HPV/p16 positive and HPV/p16 negative OPSCC, with corresponding zoom-in insets. Pink arrows indicate CD11b⁺CD14⁺HLA-DR^low/−^ cells and yellow arrows CD11b⁺CD15⁺HLA-DR^low/−^ cells. B.-G. Corresponding individual marker channels of the same fields of view. H. Corresponding phenotype maps of the same fields of view illustrating the automated classification of immune cell populations.

Additional file 3: Supp. Figure 3 Description of data: A. and C. Low-magnification composite images of multiplex immunofluorescence staining showing immune cell markers in the primary tumor and the lymph node metastases, with corresponding zoom-in insets. In A, blue arrows indicate CD68⁺CD206⁺ cells and orange arrows CD68⁺iNOS⁺ cells. In C, pink arrows indicate CD11b⁺CD14⁺HLA-DR^low/−^CD15^−^ cells and yellow arrows CD11b⁺CD15⁺HLA-DR^low/−^ cells. B. and D. Corresponding phenotype maps of the same fields of view illustrating the automated classification of immune cell populations.

Additional file 4: Supp. Figure 4 Description of data: A.–C. Non compartment-specific Kaplan–Meier curves for overall survival according to immune cell composition in the entire cohort (A), HPV/p16 positive patients (B), and HPV/p16 negative patients (C). CD11b⁺CD14⁺ is an abbreviation for the full phenotype CD11b⁺CD14⁺HLA-DR^low/−^CD15⁻.

Additional file 5: Supp. Figure 5 Description of data: A. Low-magnification composite images of multiplex immunofluorescence staining showing tumors with high or low PMN-MDSC infiltration, with corresponding zoom-in insets. Pink arrows indicate CD11b⁺CD14⁺HLA-DR^low/−^ cells and yellow arrows CD11b⁺CD14⁻HLA-DR^low/−^CD15⁺ cells. B. Corresponding phenotype maps of the same fields of view illustrating the automated classification of immune cell populations.

Additional file 6: Supp. Table S1 Title of data: Predefined gene sets of immune pathways by NanoString nSolver v4.0 (Advanced Analysis Module v2.0.134).

Additional file 7: Supp. Table S2 Title of data: Results of differential expression testing of all significantly different mRNAs in the myeloid compartment, comparing HPV/p16+ and HPV/p16- cases (baseline HPV/p16+ cases).

Additional file 8: Supp. Table S3 Title of data: Results of differential expression testing of all significantly different mRNAs in the lymphoid compartment, comparing HPV/p16+ and HPV/p16- cases (baseline HPV/p16+ cases).

Additional file 9: Supp. Table S4 Title of data: Results of differential expression testing of all significantly different mRNAs in antigen presentation pathways, comparing HPV/p16+ and HPV/p16- cases (baseline HPV/p16+ cases).

Additional file 10: Supp. Table S5 Title of data: Divergent distribution of immune cells between primary tumor and lymph node metastases.

Additional file 11: Supp. Table S6 Title of data: Divergent distribution of immune cells between the tumor and stroma compartment in HPV/p16+ and HPV/p16- cases in the primary tumor.

Additional file 12: Supp. Table S7 Title of data: Divergent distribution of immune cells between the tumor and stroma compartment in HPV/p16+ and HPV/p16- cases in the lymph node metastases.

Additional file 13: Supp. Table S8 Title of data: Univariate analysis of immune cells associated with overall survival.

## Data Availability

The datasets analysed during the current study are available from the corresponding author on reasonable request.

## Abbreviations

DEGs: Differentially expressed genes
ECS: Extracapsular spread
FFPE: Formalin fixed, paraffin embedded
FOV: Field of view
HNSCCs: Head and neck squamous cell carcinomas
HPV: Human papillomavirus
IHC: Immunohistochemistry
LM: Lymph node metastases
MDSCs: Myeloid derived suppressor cells
M-MDSCs: Monocytic myeloid-derived suppressor cells
OPSCC: Oropharyngeal squamous cell carcinoma
OS: Overall survival
PMN-MDSCs: Polymorphonuclear myeloid-derived suppressor cells
PT: Primary tumor
RNA-ISH: RNA in situ hybridization
SCC: Squamous cell carcinomas
SD: Standard deviation
TAMs: Tumor associated macrophages
TILs: Tumor infiltrating lymphocytes
TMA: Tissue microarray
TME: Tumor microenvironment
UICC: Union for International Cancer Control
