# Supplementary figures and images for: Divergent myeloid and lymphoid immune landscapes in HPV/p16 positive and HPV/p16 negative oropharyngeal squamous cell carcinomas and their lymph node metastases
Source: Mol Med. 2026 Apr 30;32:66. doi: 10.1186/s10020-026-01481-w (PMC13130499; doi:10.1186/s10020-026-01481-w)

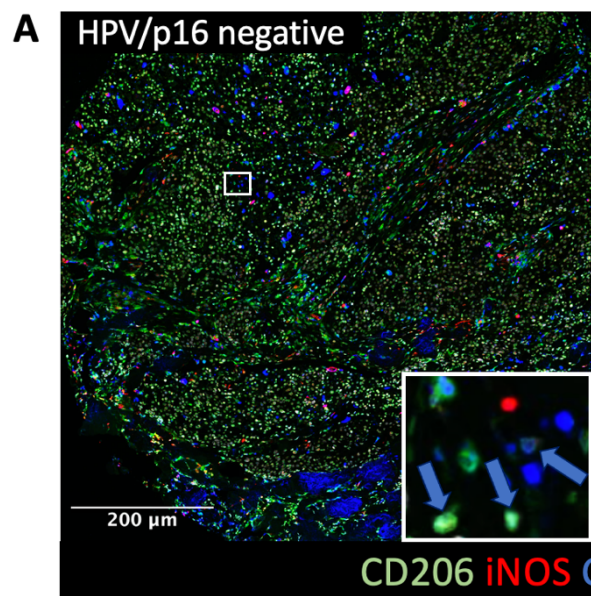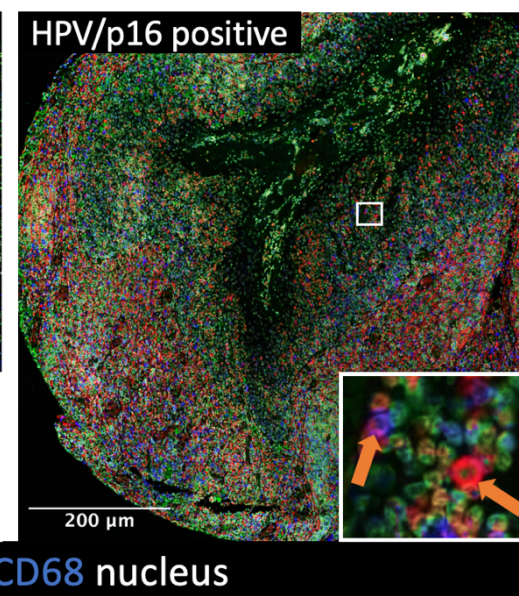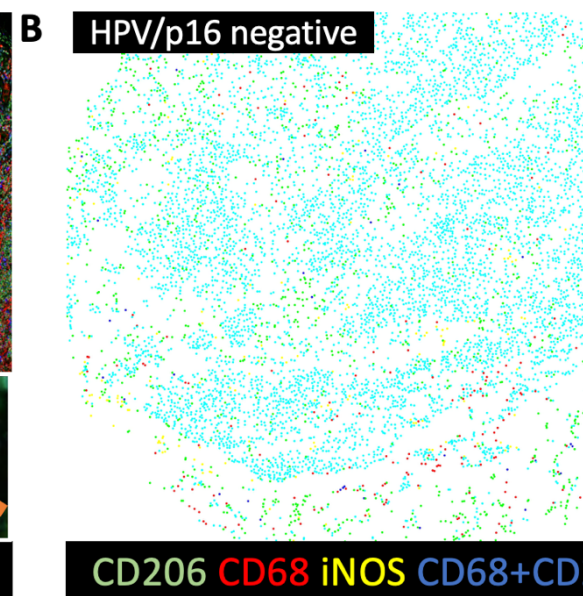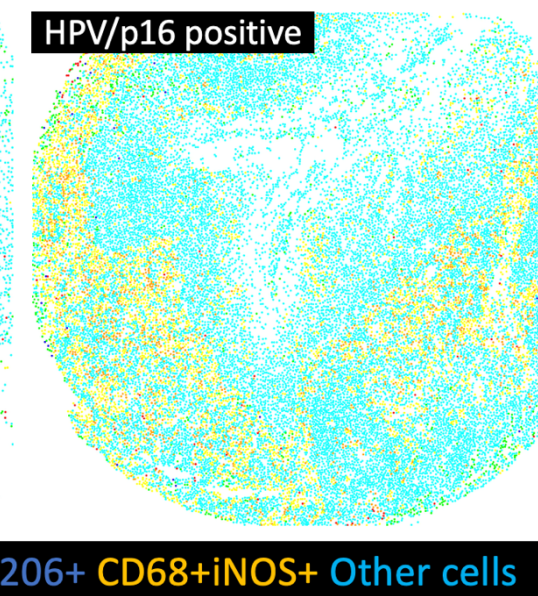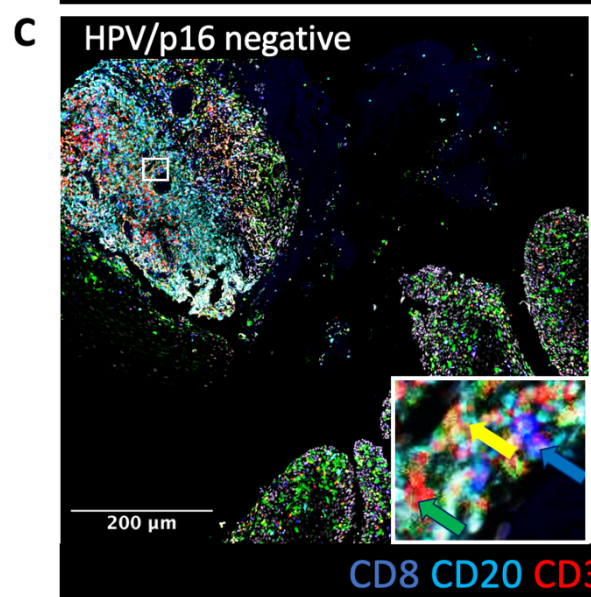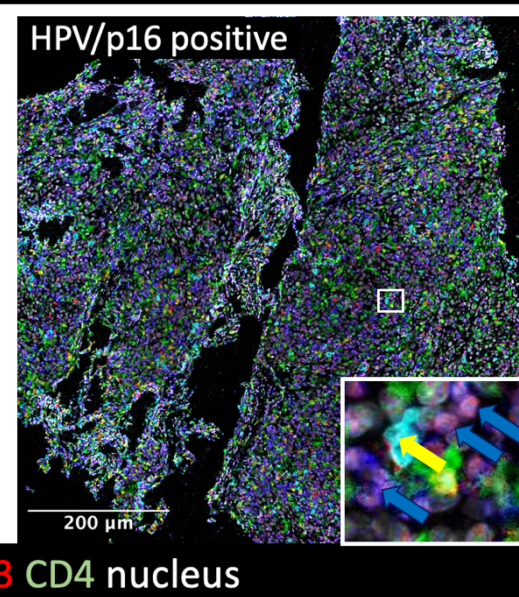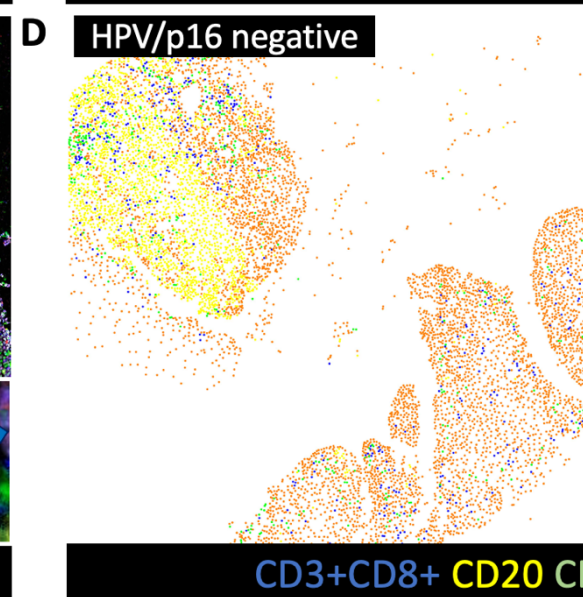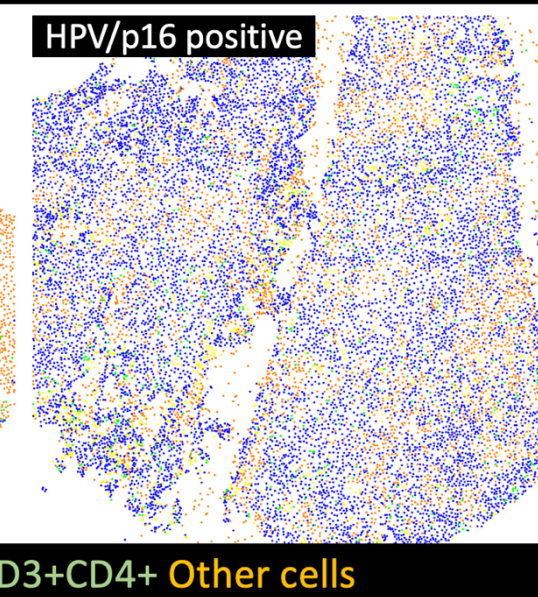

Supplement: Supplementary file 1 — Additional file 1: Supp. Figure 1 Description of data: A. and C. Low-magnification composite images of multiplex immunofluorescence staining showing immune cell markers in HPV/p16 positive and HPV/p16 negative OPSCC, with corresponding zoom-in insets. In A, blue arrows indicate CD68⁺CD206⁺ cells, and orange arrows CD68⁺iNOS⁺ cells. In C, blue arrows indicate CD3⁺CD8⁺ cells, yellow arrows CD20⁺ cells, and green arrows CD3⁺CD4⁺ cells. B. and D. Corresponding phenotype maps of the same fields of view illustrating the automated classification of immune cell populations. [file 10020_2026_1481_MOESM1_ESM.pdf]

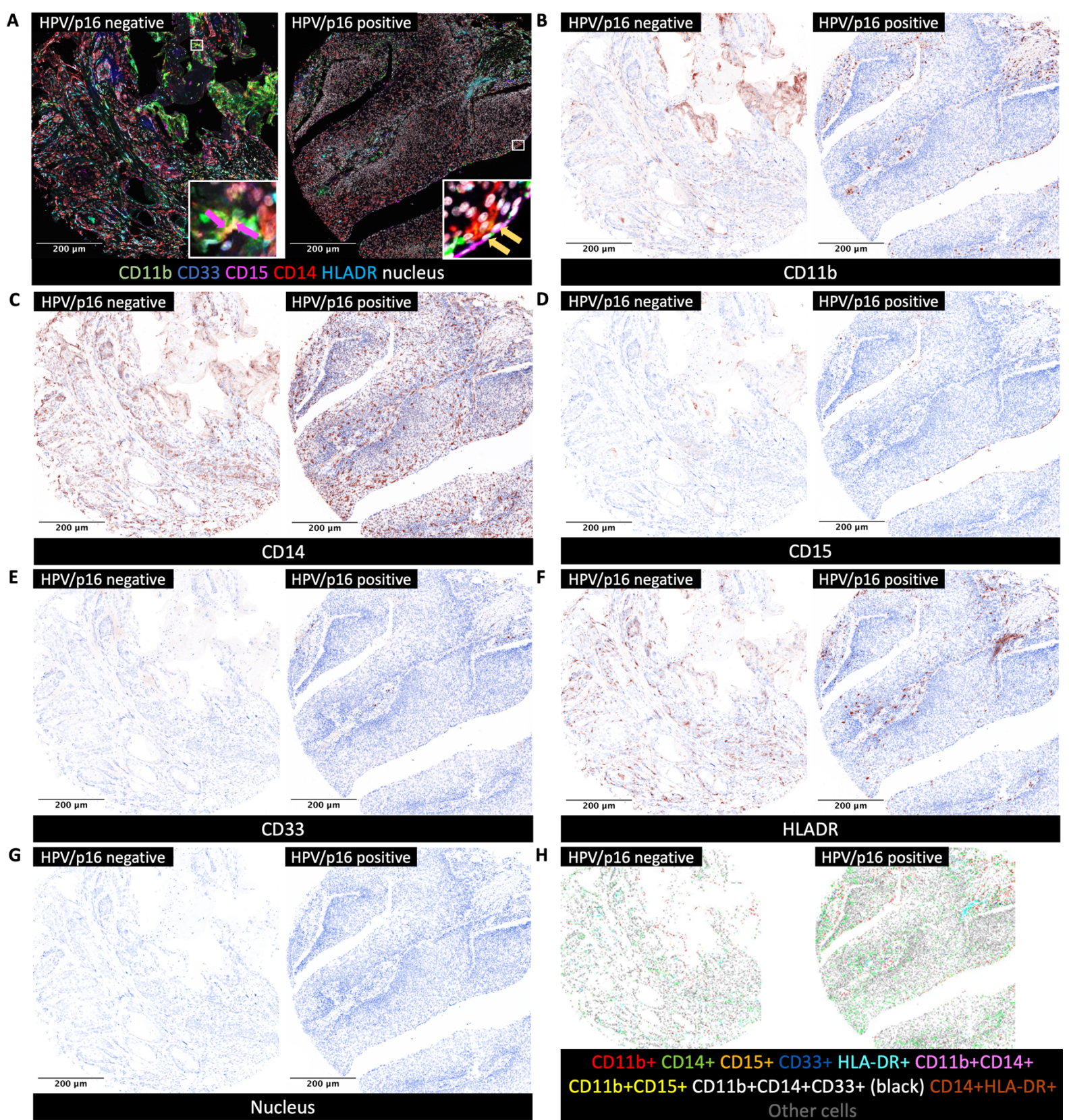

Supplement: Supplementary file 2 — Additional file 2: Supp. Figure 2 Description of data: A. Low-magnification composite images of multiplex immunofluorescence staining showing immune cell markers in HPV/p16 positive and HPV/p16 negative OPSCC, with corresponding zoom-in insets. Pink arrows indicate CD11b⁺CD14⁺HLA-DRlow/− cells and yellow arrows CD11b⁺CD15⁺HLA-DRlow/− cells. B.-G. Corresponding individual marker channels of the same fields of view. H. Corresponding phenotype maps of the same fields of view illustrating the automated classification of immune cell populations. [file 10020_2026_1481_MOESM2_ESM.pdf]

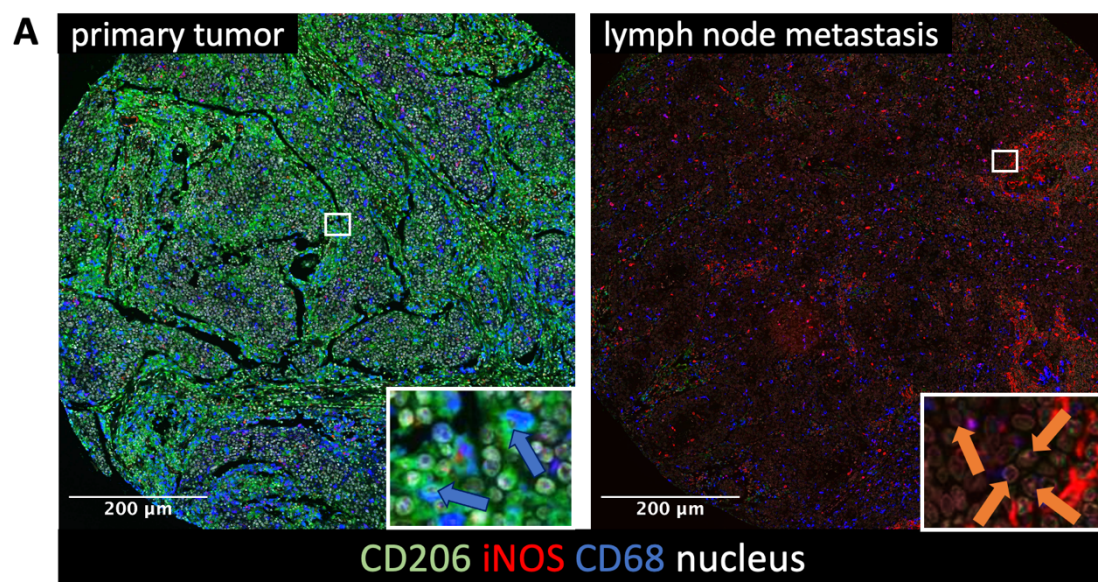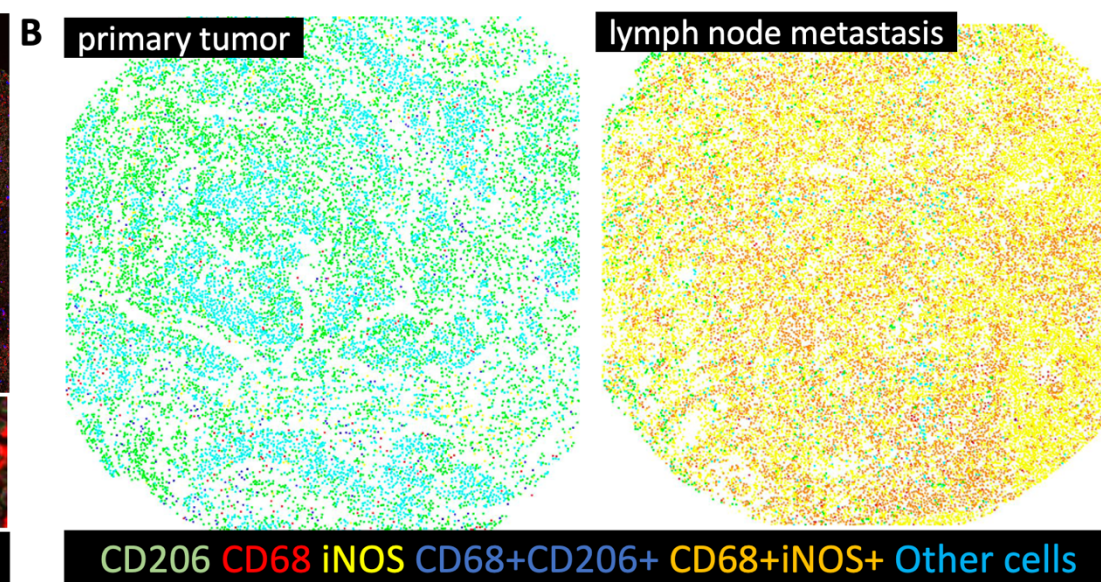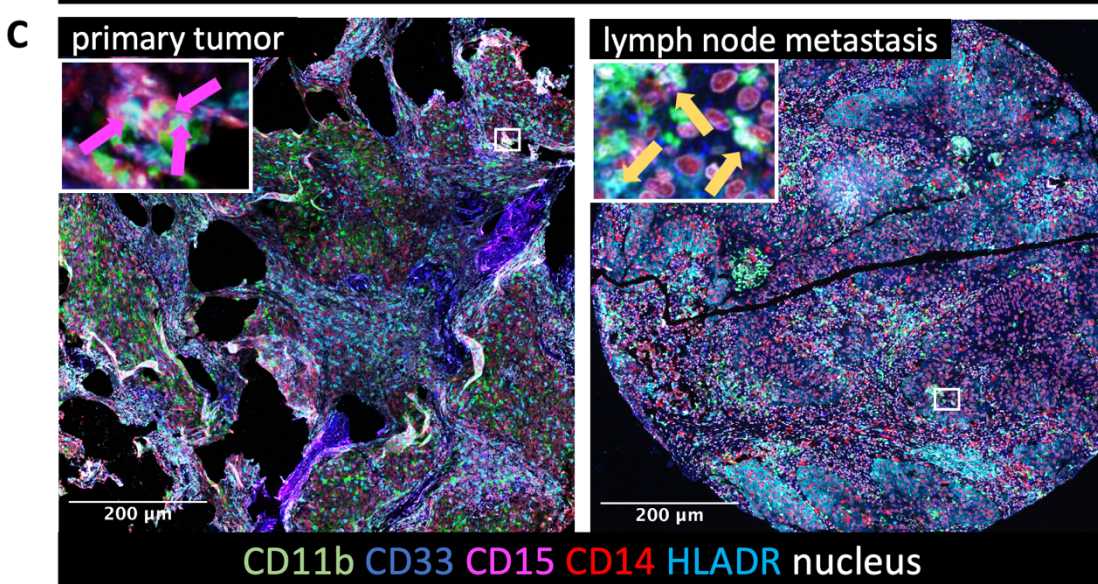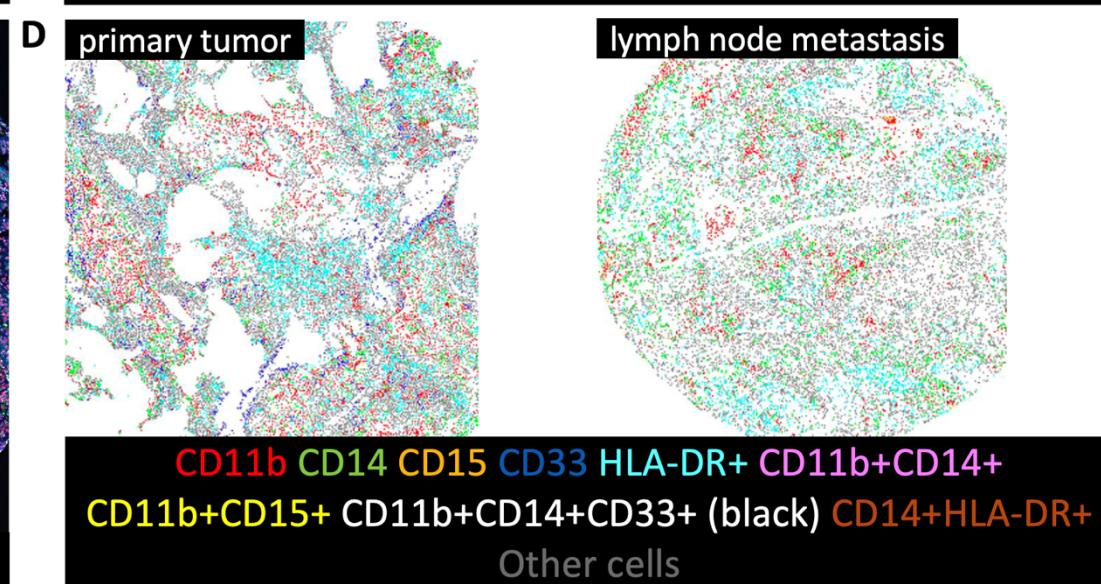

Supplement: Supplementary file 3 — Additional file 3: Supp. Figure 3 Description of data: A. and C. Low-magnification composite images of multiplex immunofluorescence staining showing immune cell markers in the primary tumor and the lymph node metastases, with corresponding zoom-in insets. In A, blue arrows indicate CD68⁺CD206⁺ cells and orange arrows CD68⁺iNOS⁺ cells. In C, pink arrows indicate CD11b⁺CD14⁺HLA-DRlow/−CD15− cells and yellow arrows CD11b⁺CD15⁺HLA-DRlow/− cells. B. and D. Corresponding phenotype maps of the same fields of view illustrating the automated classification of immune cell populations. [file 10020_2026_1481_MOESM3_ESM.pdf]

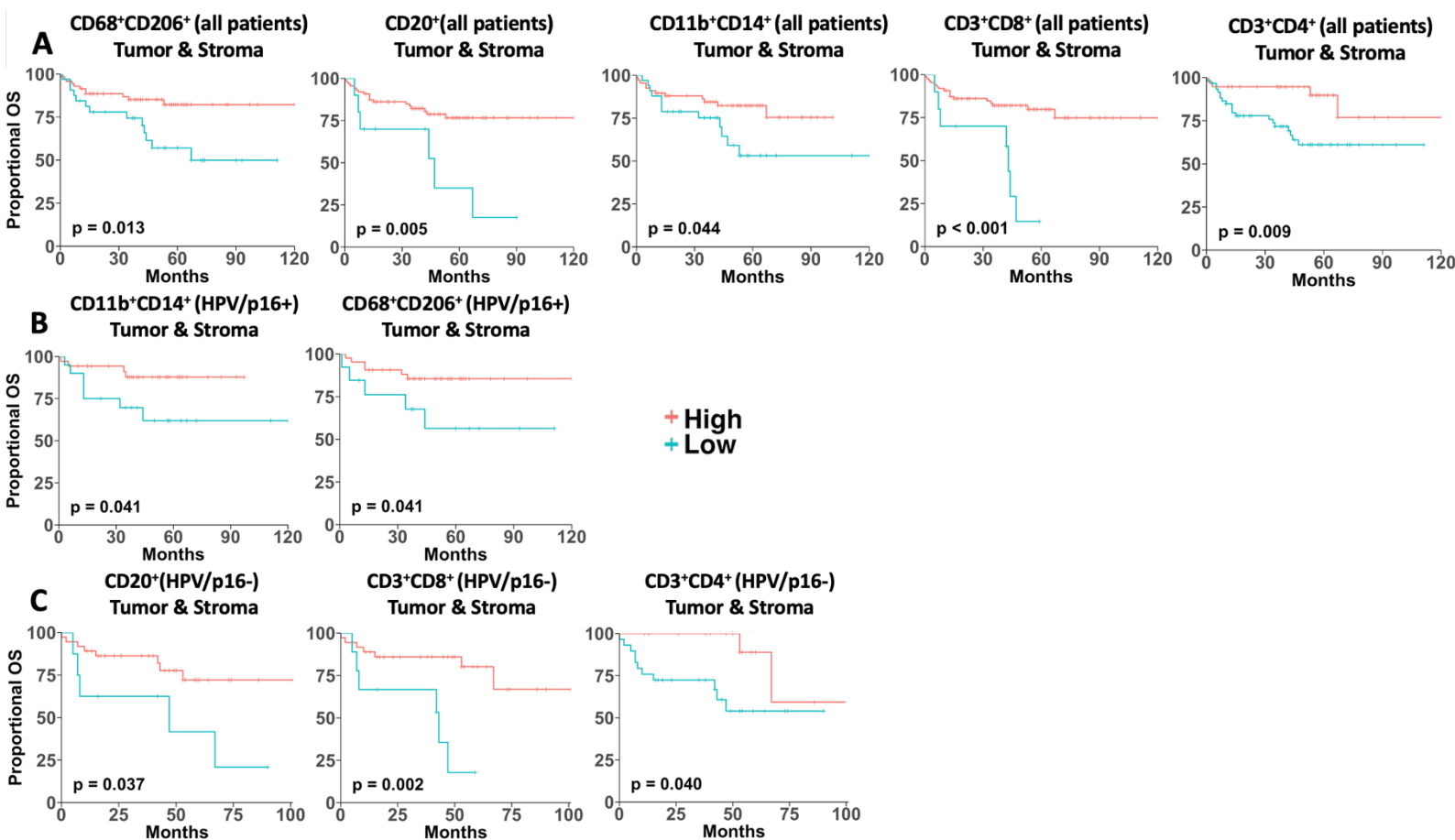

Supplement: Supplementary file 4 — Additional file 4: Supp. Figure 4 Description of data: A.–C. Non compartment-specific Kaplan–Meier curves for overall survival according to immune cell composition in the entire cohort (A), HPV/p16 positive patients (B), and HPV/p16 negative patients (C). CD11b⁺CD14⁺ is an abbreviation for the full phenotype CD11b⁺CD14⁺HLA-DRlow/−CD15⁻. [file 10020_2026_1481_MOESM4_ESM.pdf]

A

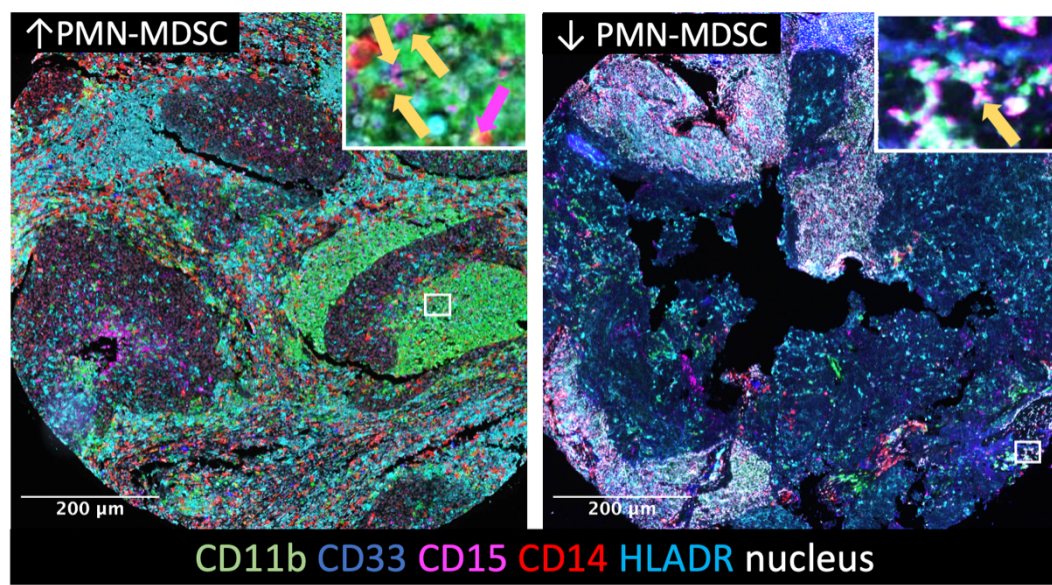

B

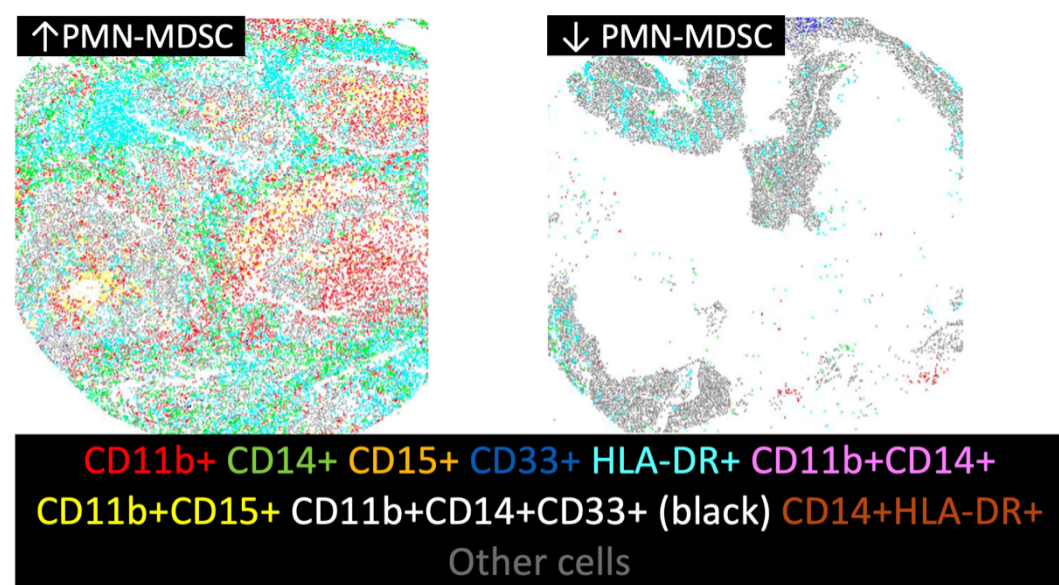

Supplement: Supplementary file 5 — Additional file 5: Supp. Figure 5 Description of data: A. Low-magnification composite images of multiplex immunofluorescence staining showing tumors with high or low PMN-MDSC infiltration, with corresponding zoom-in insets. Pink arrows indicate CD11b⁺CD14⁺HLA-DRlow/− cells and yellow arrows CD11b⁺CD14⁻HLA-DRlow/−CD15⁺ cells. B. Corresponding phenotype maps of the same fields of view illustrating the automated classification of immune cell populations. [file 10020_2026_1481_MOESM5_ESM.pdf]
