# Supplementary material for: Divergent myeloid and lymphoid immune landscapes in HPV/p16 positive and HPV/p16 negative oropharyngeal squamous cell carcinomas and their lymph node metastases
Source: Mol Med. 2026 Apr 30;32:66. doi: 10.1186/s10020-026-01481-w (PMC13130499; doi:10.1186/s10020-026-01481-w)
Supplement: Supplementary file 6 — Additional file 6: Supp. Table S1 Title of data: Predefined gene sets of immune pathways by NanoString nSolver v4.0 (Advanced Analysis Module v2.0.134). [file 10020_2026_1481_MOESM6_ESM.docx]

**Supp. Table S1.** Predefined gene sets of immune pathways by NanoString nSolver v4.0 (Advanced Analysis Module v2.0.134).

| **mRNAs of the lymphoid compartment** | | | |
| --- | --- | --- | --- |
| IFIT1-mRNA | MX1-mRNA | CXCL11-mRNA | EGR1-mRNA |
| CD40-mRNA | CD19-mRNA | CXCL9-mRNA | GZMH-mRNA |
| ISG15-mRNA | CD79B-mRNA | CD38-mRNA | CD7-mRNA |
| CD8B-mRNA | KLRB1-mRNA | CD70-mRNA | CD79A-mRNA |
| ICOSLG-mRNA | CD8A-mRNA | KLRD1-mRNA | LAG3-mRNA |
| ZAP70-mRNA | CD1C-mRNA | GZMA-mRNA | IDO1-mRNA |
| CD3G-mRNA | CD5-mRNA | CD80-mRNA | CXCR3-mRNA |
| PVR-mRNA | CD6-mRNA | GNLY-mRNA | CD274-mRNA |
| IL2RG-mRNA | ICOS-mRNA | IFNG-mRNA | CD40LG-mRNA |
| IFI27-mRNA | IL12RB2-mRNA | SLAMF7-mRNA | CXCL10-mRNA |
| CD96-mRNA | TIGIT-mRNA | PRF1-mRNA | LY9-mRNA |
| IL11-mRNA | KLRK1-mRNA | ITGA1-mRNA | EOMES-mRNA |
| F2RL1-mRNA | CD27-mRNA | IFITM1-mRNA | STAT1-mRNA |
| JAK2-mRNA | DPP4-mRNA | IRF4-mRNA | IL18R1-mRNA |
| CD3D-mRNA | IGF2R-mRNA | TNFRSF25-mRNA | BLK-mRNA |
| HLA-DOB-mRNA | CD48-mRNA | IRF9-mRNA | PDCD1-mRNA |
| LCK-mRNA | GZMK-mRNA | GZMB-mRNA | CD2-mRNA |
| CX3CL1-mRNA | CTLA4-mRNA | STAT2-mRNA | CCR4-mRNA |
| MS4A1-mRNA | CXCL13-mRNA | CD86-mRNA |  |
| CD28-mRNA | CXCL16-mRNA | TBX21-mRNA |  |
| CD3E-mRNA | GZMM-mRNA | JAK1-mRNA |  |
| **mRNAs of the myeloid compartment** | | | |
| AREG-mRNA | SIRPB2-mRNA | SIRPA-mRNA | OLR1-mRNA |
| CCL20-mRNA | CSF1-mRNA | MARCO-mRNA | CD14-mRNA |
| S100A12-mRNA | CD47-mRNA | CCL5-mRNA | LAMB3-mRNA |
| FOSL1-mRNA | CLEC7A-mRNA | FPR3-mRNA | CLEC5A-mRNA |
| CRABP2-mRNA | P2RY13-mRNA | CSF1R-mRNA | CXCL6-mRNA |
| LYZ-mRNA | IL1RN-mRNA | TLR4-mRNA | TLR2-mRNA |
| MMP1-mRNA | S100A9-mRNA | NLRP3-mRNA | LIF-mRNA |
| TREM1-mRNA | CXCL12-mRNA | CYBB-mRNA | CXCL5-mRNA |
| CSF3R-mRNA | LILRB2-mRNA | SERPINA1-mRNA | FCAR-mRNA |
| CEBPB-mRNA | S100A8-mRNA | ITGAM-mRNA | IL1B-mRNA |
| SLC11A1-mRNA | CXCL2-mRNA | TLR8-mRNA | CCL8-mRNA |
| IER3-mRNA | PDZK1IP1-mRNA | CCL4-mRNA | CDKN1A-mRNA |
| PTGS2-mRNA | DLL4-mRNA | LY96-mRNA | MRC1-mRNA |
| TNFAIP6-mRNA | CXCL3-mRNA | FCN1-mRNA | IL1A-mRNA |
| COL17A1-mRNA | COL11A1-mRNA | HCK-mRNA | DAB2-mRNA |
| TLR1-mRNA | CXCL1-mRNA | NFAM1-mRNA | TREM2-mRNA |
| LILRA5-mRNA | CCL2-mRNA | ITGAX-mRNA | C5AR1-mRNA |
| FPR1-mRNA | FCGR1A-mRNA | P2RY13-mRNA | ANGPT1-mRNA |
| **mRNAs of antigen presentation** | | | |
| PSMB5-mRNA | ITGAV-mRNA | HLA-DMA-mRNA | HLA-E-mRNA |
| CD36-mRNA | UBA7-mRNA | HLA-A-mRNA | HLA-DQB1-mRNA |
| HLA-DQA2-mRNA | CTSW-mRNA | CD4-mRNA | TAP1-mRNA |
| CD8B-mRNA | HLA-DMB-mRNA | HLA-B-mRNA | HLA-F-mRNA |
| THBD-mRNA | TNF-mRNA | TRIM21-mRNA | CDC20-mRNA |
| HLA-DOB-mRNA | CCR5-mRNA | SOCS1-mRNA | PSMB8-mRNA |
| HLA-DRA-mRNA | ULBP2-mRNA | CTSS-mRNA | TAP2-mRNA |
| CD8A-mRNA | IRF8-mRNA | CYBB-mRNA | FCGR1A-mRNA |
| CD1C-mRNA | DTX3L-mRNA | IFNG-mRNA | ATF3-mRNA |
| TAPBP-mRNA | HLA-DRB1-mRNA | UBE2C-mRNA | BATF3-mRNA |
| HLA-DOA-mRNA | CXCL1-mRNA | HLA-C-mRNA | HLA-DPA1-mRNA |
| PSMB10-mRNA | PSMB9-mRNA | B2M-mRNA | MRC1-mRNA |
| KIF2C-mRNA | KLRD1-mRNA | HLA-DQA1-mRNA | CD74-mRNA |
| HLA-DPB1-mRNA | VHL-mRNA | CCL4-mRNA |  |
| **Macrophages** | | | |
| CD68-mRNA | CD84-mRNA | MS4A4A-mRNA | CD163-mRNA |
| **Exhausted CD8 T cells** | | | |
| CD244-mRNA | LAG3-mRNA | PTGER4-mRNA |  |
| **B cells** | | | |
| FCRL2-mRNA | TNFRSF17-mRNA | PNOC-mRNA | MS4A1-mRNA |
| TCL1A-mRNA | CD19-mRNA | SPIB-mRNA | BLK-mRNA |
| FAM30A-mRNA |  |  |  |
| **CD45 cells** | | | |
| CD45RA-mRNA | PTPRC-mRNA | CD45RO-mRNA |  |
| **T cells** | | | |
| TRAT1-mRNA | CD3D-mRNA | CD3G-mRNA | CD6-mRNA |
| CD3E-mRNA | SH2D1A-mRNA |  |  |
| **CD8 T cells** | | | |
| CD8A-mRNA | CD8B-mRNA |  |  |
