# Supplementary material for: Divergent myeloid and lymphoid immune landscapes in HPV/p16 positive and HPV/p16 negative oropharyngeal squamous cell carcinomas and their lymph node metastases
Source: Mol Med. 2026 Apr 30;32:66. doi: 10.1186/s10020-026-01481-w (PMC13130499; doi:10.1186/s10020-026-01481-w)
Supplement: Supplementary file 7 — Additional file 7: Supp. Table S2 Title of data: Results of differential expression testing of all significantly different mRNAs in the myeloid compartment, comparing HPV/p16+ and HPV/p16- cases (baseline HPV/p16+ cases). [file 10020_2026_1481_MOESM7_ESM.docx]

**Supp. Table S2.** Results of differential expression testing of all significantly different mRNAs in the myeloid compartment, comparing HPV/p16+ and HPV/p16- cases (baseline HPV/p16+ cases).

| **mRNA** | **Log2 fold change** | **Lower confidence limit (log2)** | **Upper confidence limit (log2)** | **P value** | **FDR P value** |
| --- | --- | --- | --- | --- | --- |
| **AREG-mRNA** | 2.02 | 1.44 | 2.6 | 1.61E-09 | 2.03E-06 |
| **CCL20-mRNA** | -1.79 | -2.45 | -1.14 | 7.97E-07 | 0.000268 |
| **S100A12-mRNA** | 1.29 | 0.776 | 1.81 | 4.72E-06 | 0.00104 |
| **FOSL1-mRNA** | 1.39 | 0.823 | 1.96 | 6.93E-06 | 0.00117 |
| **CRABP2-mRNA** | 1.14 | 0.656 | 1.62 | 1.34E-05 | 0.00211 |
| **LYZ-mRNA** | 1.49 | 0.853 | 2.12 | 1.49E-05 | 0.00221 |
| **MMP1-mRNA** | 1.73 | 0.902 | 2.55 | 9.61E-05 | 0.00951 |
| **TREM1-mRNA** | 1.06 | 0.54 | 1.57 | 0.000133 | 0.0125 |
| **CSF3R-mRNA** | 0.774 | 0.369 | 1.18 | 0.00033 | 0.0249 |
| **CEBPB-mRNA** | 0.718 | 0.337 | 1.1 | 0.000403 | 0.0275 |
| **SLC11A1-mRNA** | 0.96 | 0.44 | 1.48 | 0.000513 | 0.0312 |
| **IER3-mRNA** | 0.95 | 0.417 | 1.48 | 0.000767 | 0.0418 |
| **PTGS2-mRNA** | 0.809 | 0.352 | 1.27 | 0.000839 | 0.0436 |
| **TNFAIP6-mRNA** | 0.947 | 0.38 | 1.51 | 0.00154 | 0.0714 |
| **COL17A1-mRNA** | 0.967 | 0.367 | 1.57 | 0.0022 | 0.0905 |
| **TLR1-mRNA** | -0.513 | -0.832 | -0.194 | 0.00225 | 0.0905 |
| **LILRA5-mRNA** | 0.803 | 0.297 | 1.31 | 0.00254 | 0.0964 |
| **FPR1-mRNA** | 0.694 | 0.256 | 1.13 | 0.00261 | 0.0981 |
| **TLR2-mRNA** | 0.301 | 0.103 | 0.498 | 0.00378 | 0.122 |
| **LIF-mRNA** | 0.84 | 0.271 | 1.41 | 0.00488 | 0.142 |

FDR P value, False Discovery Rate adjusted P value.
