# Supplementary material for: Divergent myeloid and lymphoid immune landscapes in HPV/p16 positive and HPV/p16 negative oropharyngeal squamous cell carcinomas and their lymph node metastases
Source: Mol Med. 2026 Apr 30;32:66. doi: 10.1186/s10020-026-01481-w (PMC13130499; doi:10.1186/s10020-026-01481-w)
Supplement: Supplementary file 8 — Additional file 8: Supp. Table S3 Title of data: Results of differential expression testing of all significantly different mRNAs in the lymphoid compartment, comparing HPV/p16+ and HPV/p16- cases (baseline HPV/p16+ cases). [file 10020_2026_1481_MOESM8_ESM.docx]

**Supp. Table S3.** Results of differential expression testing of all significantly different mRNAs in the lymphoid compartment, comparing HPV/p16+ and HPV/p16- cases (baseline HPV/p16+ cases).

| **mRNA** | **Log2 fold change** | **Lower confidence limit (log2)** | **Upper confidence limit (log2)** | **P value** | **FDR P value** |
| --- | --- | --- | --- | --- | --- |
| **IFIT1-mRNA** | 1.05 | 0.674 | 1.42 | 4.35E-07 | 0.000194 |
| **CD40-mRNA** | -0.568 | -0.774 | -0.363 | 5.74E-07 | 0.000223 |
| **ISG15-mRNA** | 0.973 | 0.579 | 1.37 | 6.02E-06 | 0.0011 |
| **CD8B-mRNA** | -1.28 | -1.8 | -0.76 | 6.3E-06 | 0.0011 |
| **ICOSLG-mRNA** | -0.604 | -0.878 | -0.33 | 4.25E-05 | 0.00487 |
| **ZAP70-mRNA** | -0.889 | -1.33 | -0.452 | 0.000141 | 0.0127 |
| **CD3G-mRNA** | -0.708 | -1.06 | -0.352 | 0.000194 | 0.0163 |
| **PVR-mRNA** | 0.732 | 0.344 | 1.12 | 0.000383 | 0.0273 |
| **IL2RG-mRNA** | -0.811 | -1.24 | -0.38 | 0.000401 | 0.0275 |
| **IFI27-mRNA** | 0.719 | 0.334 | 1.1 | 0.000436 | 0.0285 |
| **CD96-mRNA** | -0.698 | -1.07 | -0.323 | 0.000457 | 0.0292 |
| **IL11-mRNA** | 1.29 | 0.578 | 2 | 0.000623 | 0.0366 |
| **F2RL1-mRNA** | 1.17 | 0.523 | 1.82 | 0.000649 | 0.0372 |
| **JAK2-mRNA** | -0.457 | -0.711 | -0.204 | 0.000664 | 0.0377 |
| **CD3D-mRNA** | -0.731 | -1.14 | -0.32 | 0.000793 | 0.0423 |
| **HLA-DOB-mRNA** | -0.733 | -1.15 | -0.319 | 0.000823 | 0.0433 |

FDR P value, False Discovery Rate adjusted P value.
