# Supplementary material for: Divergent myeloid and lymphoid immune landscapes in HPV/p16 positive and HPV/p16 negative oropharyngeal squamous cell carcinomas and their lymph node metastases
Source: Mol Med. 2026 Apr 30;32:66. doi: 10.1186/s10020-026-01481-w (PMC13130499; doi:10.1186/s10020-026-01481-w)
Supplement: Supplementary file 9 — Additional file 9: Supp. Table S4 Title of data: Results of differential expression testing of all significantly different mRNAs in antigen presentation pathways, comparing HPV/p16+ and HPV/p16- cases (baseline HPV/p16+ cases). [file 10020_2026_1481_MOESM9_ESM.docx]

**Supp. Table S4.** Results of differential expression testing of all significantly different mRNAs in antigen presentation pathways, comparing HPV/p16+ and HPV/p16- cases (baseline HPV/p16+ cases).

| **mRNA** | **Log2 fold change** | **Lower confidence limit (log2)** | **Upper confidence limit (log2)** | **P value** | **FDR P value** |
| --- | --- | --- | --- | --- | --- |
| **PSMB5-mRNA** | 0.485 | 0.311 | 0.659 | 4.61E-07 | 0.000194 |
| **CD36-mRNA** | 1.21 | 0.766 | 1.65 | 7.31E-07 | 0.000264 |
| **HLA-DQA2-mRNA** | -2.02 | -2.84 | -1.2 | 6.25E-06 | 0.0011 |
| **CD8B-mRNA** | -1.28 | -1.8 | -0.76 | 6.3E-06 | 0.0011 |
| **THBD-mRNA** | 0.833 | 0.441 | 1.23 | 7.66E-05 | 0.00805 |
| **HLA-DOB-mRNA** | -0.733 | -1.15 | -0.319 | 0.000823 | 0.0433 |

FDR P value, False Discovery Rate adjusted P value.
