# Supplementary material for: Divergent myeloid and lymphoid immune landscapes in HPV/p16 positive and HPV/p16 negative oropharyngeal squamous cell carcinomas and their lymph node metastases
Source: Mol Med. 2026 Apr 30;32:66. doi: 10.1186/s10020-026-01481-w (PMC13130499; doi:10.1186/s10020-026-01481-w)
Supplement: Supplementary file 10 — Additional file 10: Supp. Table S5 Title of data: Divergent distribution of immune cells between primary tumor and lymph node metastases. [file 10020_2026_1481_MOESM10_ESM.docx]

**Supp. Table S5.** Divergent distribution of immune cells between primary tumor and lymph node metastases.

| **HPV/p16+** | | | | |
| --- | --- | --- | --- | --- |
| **Variables** | **N** | **Primary tumor**  **Median (range)** | **Lymph node metastasis**  **Median (range)** | **P value** |
| **Total** | | | | |
| **CD68+CD206+** | 34/33 | 27.00 (199.00) | 6.25 (70.50) | **<0.001** |
| **CD68+iNOS+** | 34/33 | 25.25 (678.00) | 856.50 (8448.50) | **<0.001** |
| **CD11b+CD14+**^†^ | 34/33 | 4.00 (171.00) | 0.50 (12.50) | **<0.001** |
| **CD11b+CD15+**^†^ | 34/33 | 1.75 (61.00) | 15.00 (448.50) | **<0.001** |
| **CD3+CD4+** | 32/29 | 442.75 (2802.00) | 294 .00 (5380.00) | 0.400 |
| **CD3+CD8+** | 32/29 | 1905.25 (7523.00) | 1996.00 (6678.50) | 0.517 |
| **CD20+** | 32/29 | 1091.25 (4426.00) | 810.50 (8347.50) | 0.597 |
| **Tumor compartment** | | | | |
| **CD68+CD206+** | 34/34 | 13.50 (102.50) | 2.25 (25.00) | **<0.001** |
| **CD68+iNOS+** | 34/34 | 23.00 (678.50) | 662.25 (8284.50) | **<0.001** |
| **CD11b+CD14+**^†^ | 34/33 | 1.00 (20.00) | 0.00 (11.00) | **0.025** |
| **CD11b+CD15+**^†^ | 34/33 | 0.00 (11.50) | 11.00 (446.50) | **<0.001** |
| **CD3+CD4+** | 32/29 | 154.25 (416.00) | 127.50 (334.50) | 0.056 |
| **CD3+CD8+** | 32/29 | 1056.50 (3003.00) | 705.50 (4504.50) | 0.572 |
| **CD20+** | 32/29 | 521.50 (2818.00) | 656.00 (7021.50) | 0.079 |
| **Stroma compartment** | | | | |
| **CD68+CD206+** | 34/34 | 13.75 (131.00) | 2.75 (47.00) | **<0.001** |
| **CD68+iNOS+** | 34/34 | 1.00 (21.50) | 133.50 (1560.50) | **<0.001** |
| **CD11b+CD14+**^†^ | 34/33 | 2.75 (159.50) | 0.00 (1.50) | **<0.001** |
| **CD11b+CD15+**^†^ | 34/33 | 1.50 (49.50) | 0.50 (43.50) | 0.166 |
| **CD3+CD4+** | 32/29 | 244.25 (2422.50) | 163.50 (5323.00) | 0.639 |
| **CD3+CD8+** | 32/29 | 775.50 (5105.50) | 598.50 (2493.00) | 0.220 |
| **CD20+** | 32/29 | 453.75 (2794.00) | 264.00 (1329.50) | **0.046** |
| **HPV/p16-** | | | | |
| **Variables** | **N** | **Primary tumor**  **Median (range)** | **Lymph node metastasis**  **Median (range)** | **P value** |
| **Total** | | | | |
| **CD68+CD206+** | 26/24 | 14.75 (189.00) | 5.25 (55.50) | **0.003** |
| **CD68+iNOS+** | 26/24 | 4.00 (168.50) | 427.50 (6183.00) | **<0.001** |
| **CD11b+CD14+**^†^ | 26/26 | 7.25 (88.50) | 0.50 (41.00) | **<0.001** |
| **CD11b+CD15+**^†^ | 26/26 | 4.00 (113.00) | 29.00 (1538.50) | **<0.001** |
| **CD3+CD4+** | 25/25 | 386.50 (805.50) | 391.00 (1626.50) | 0.230 |
| **CD3+CD8+** | 25/25 | 561.00 (10545.00) | 834.50 (11909.50) | **0.016** |
| **CD20+** | 25/25 | 599.50 (4644.00) | 516.00 (9997.00) | 0.568 |
| **Tumor compartment** | | | | |
| **CD68+CD206+** | 26/24 | 6.50 (87.50) | 2.25 (39.00) | **0.036** |
| **CD68+iNOS+** | 26/24 | 2.25 (148.00) | 296.00 (7425.00) | **<0.001** |
| **CD11b+CD14+**^†^ | 26/26 | 0.50 (13.50) | 0.00 (40.50) | 0.305 |
| **CD11b+CD15+**^†^ | 26/26 | 0.50 (33.00) | 28.25 (1531.50) | **<0.001** |
| **CD3+CD4+** | 25/25 | 105.50 (411.50) | 99.00 (460.00) | 0.775 |
| **CD3+CD8+** | 25/25 | 223.50 (4274.50) | 698.00 (11344.00) | **0.010** |
| **CD20+** | 25/25 | 193.00 (4585.50) | 270.50 (8682.50) | 0.440 |
| **Stroma compartment** | | | | |
| **CD68+CD206+** | 26/24 | 9.00 (152.00) | 2.00 (46.00) | **0.001** |
| **CD68+iNOS+** | 26/24 | 1.50 (20.50) | 37.75 (565.50) | **<0.001** |
| **CD11b+CD14+**^†^ | 26/26 | 6.00 (75.00) | 0.00 (1.50) | **<0.001** |
| **CD11b+CD15+**^†^ | 26/26 | 3.00 (84.00) | 1.00 (154.00) | 0.075 |
| **CD3+CD4+** | 25/25 | 234.50 (577.50) | 269.50 (1179.50) | 0.284 |
| **CD3+CD8+** | 25/25 | 227.50 (6280.00) | 402.00 (1976.00) | 0.145 |
| **CD20+** | 25/25 | 169.50 (1564.00) | 245.50 (1319.00) | 0.855 |

† Abbreviations: CD11b⁺CD15⁺ and CD11b⁺CD14⁺ denote CD11b⁺CD14⁻HLA-DR^low/−^CD15⁺ and CD11b⁺CD14⁺HLA-DR^low/−^CD15⁻, respectively.
