# Supplementary material for: Divergent myeloid and lymphoid immune landscapes in HPV/p16 positive and HPV/p16 negative oropharyngeal squamous cell carcinomas and their lymph node metastases
Source: Mol Med. 2026 Apr 30;32:66. doi: 10.1186/s10020-026-01481-w (PMC13130499; doi:10.1186/s10020-026-01481-w)
Supplement: Supplementary file 11 — Additional file 11: Supp. Table S6 Title of data: Divergent distribution of immune cells between the tumor and stroma compartment in HPV/p16+ and HPV/p16- cases in the primary tumor. [file 10020_2026_1481_MOESM11_ESM.docx]

**Supp. Table S6.** Divergent distribution of immune cells between the tumor and stroma compartment in HPV/p16+ and HPV/p16- cases in the primary tumor.

| **HPV/p16+** | | | | |
| --- | --- | --- | --- | --- |
| **Variables** | **N** | **Tumor compartment**  **Median (range)** | **Stroma compartment**  **Median (range)** | **P value** |
| **CD68+CD206+** | 56 | 12.25 (102.50) | 10.50 (189.00) | 0.857 |
| **CD68+iNOS+** | 56 | 16.75 (707.00) | 1.00 (21.50) | **<0.001** |
| **CD11b+CD14+**^†^ | 55 | 0.50 (20.00) | 3.50 (159.50) | **<0.001** |
| **CD11b+CD15+**^†^ | 55 | 0.50 (22.00) | 2.00 (435.00) | **<0.001** |
| **CD3+CD4+** | 52 | 158.00 (629.50) | 235.25 (2422.50) | **<0.001** |
| **CD3+CD8+** | 52 | 926.25 (4081.00) | 627.50 (5105.50) | **0.014** |
| **CD20+** | 52 | 563.50 (8511.00) | 427.00 (2794.00) | **0.032** |
| **HPV/p16-** | | | | |
| **Variables** | **N** | **Tumor compartment**  **Median (range)** | **Stroma compartment**  **Median (range)** | **P value** |
| **CD68+CD206+** | 46 | 6.25 (109.00) | 10.75 (153.00) | **0.004** |
| **CD68+iNOS+** | 46 | 2.00 (179.50) | 1.25 (20.50) | **<0.001** |
| **CD11b+CD14+**^†^ | 45 | 0.50 (13.50) | 6.50 (75.00) | **<0.001** |
| **CD11b+CD15+**^†^ | 45 | 0.50 (33.00) | 3.00 (87.00) | **<0.001** |
| **CD3+CD4+** | 45 | 105.50 (433.00) | 285.00 (1439.00) | **<0.001** |
| **CD3+CD8+** | 45 | 204.50 (4280.00) | 241.50 (6280.00) | 0.343 |
| **CD20+** | 45 | 201.00 (4585.50) | 336.00 (2759.00) | 0.460 |

† Abbreviations: CD11b⁺CD15⁺ and CD11b⁺CD14⁺ denote CD11b⁺CD14⁻HLA-DR^low/−^CD15⁺ and CD11b⁺CD14⁺HLA-DR^low/−^CD15⁻, respectively.
