# Supplementary material for: Divergent myeloid and lymphoid immune landscapes in HPV/p16 positive and HPV/p16 negative oropharyngeal squamous cell carcinomas and their lymph node metastases
Source: Mol Med. 2026 Apr 30;32:66. doi: 10.1186/s10020-026-01481-w (PMC13130499; doi:10.1186/s10020-026-01481-w)
Supplement: Supplementary file 12 — Additional file 12: Supp. Table S7 Title of data: Divergent distribution of immune cells between the tumor and stroma compartment in HPV/p16+ and HPV/p16- cases in the lymph node metastases. [file 10020_2026_1481_MOESM12_ESM.docx]

**Supp. Table S7.** Divergent distribution of immune cells between the tumor and stroma compartment in HPV/p16+ and HPV/p16- cases in the lymph node metastases.

| **HPV/p16+** | | | | |
| --- | --- | --- | --- | --- |
| **Variables** | **N** | **Tumor compartment**  **Median (range)** | **Stroma compartment**  **Median (range)** | **P value** |
| **CD68+CD206+** | 29 | 2.00 (25.00) | 3.00 (47.00) | 0.080 |
| **CD68+iNOS+** | 29 | 637.00 (8284.50) | 139.00 (1558.00) | **0.002** |
| **CD11b+CD14+**^†^ | 28 | 0.00 (11.00) | 0.00 (1.50) | **0.006** |
| **CD11b+CD15+**^†^ | 28 | 15.00 (446.50) | 0.50 (43.50) | **<0.001** |
| **CD3+CD4+** | 26 | 114.94 (69.72)^1^ | 165.00 (5323.00) | **0.004** |
| **CD3+CD8+** | 26 | 692.75 (4504.50) | 561.75 (2493.00) | 0.101 |
| **CD20+** | 26 | 649.00 (7021.50) | 271.00 (1327.50) | **<0.001** |
| **HPV/p16-** | | | | |
| **Variables** | **N** | **Tumor compartment**  **Median (range)** | **Stroma compartment**  **Median (range)** | **P value** |
| **CD68+CD206+** | 23 | 2.00 (39.00) | 2.00 (46.00) | 0.952 |
| **CD68+iNOS+** | 23 | 230.00 (7425.00) | 25.00 (565.50) | **<0.001** |
| **CD11b+CD14+**^†^ | 25 | 0.00 (40.50) | 0.00 (1.50) | **0.019** |
| **CD11b+CD15+**^†^ | 25 | 32.50 (1531.50) | 1.00 (154.00) | **<0.001** |
| **CD3+CD4+** | 24 | 104.50 (448.50) | 274.75 (1179.50) | **0.002** |
| **CD3+CD8+** | 24 | 716.75 (11285.50) | 403.00 (1976.00) | 0.086 |
| **CD20+** | 24 | 308.75 (8682.50) | 239.50 (1319.00) | **0.022** |

† Abbreviations: CD11b⁺CD15⁺ and CD11b⁺CD14⁺ denote CD11b⁺CD14⁻HLA-DR^low/−^CD15⁺ and CD11b⁺CD14⁺HLA-DR^low/−^CD15⁻, respectively.
