# Supplementary material for: Divergent myeloid and lymphoid immune landscapes in HPV/p16 positive and HPV/p16 negative oropharyngeal squamous cell carcinomas and their lymph node metastases
Source: Mol Med. 2026 Apr 30;32:66. doi: 10.1186/s10020-026-01481-w (PMC13130499; doi:10.1186/s10020-026-01481-w)
Supplement: Supplementary file 13 — Additional file 13: Supp. Table S8 Title of data: Univariate analysis of immune cells associated with overall survival. [file 10020_2026_1481_MOESM13_ESM.docx]

**Supp. Table S8.** Univariate analysis of immune cells associated with overall survival.

| **Variable** | | **Total** | | | **HPV/p16+** | | | **HPV/p16-** | | |
| --- | --- | --- | --- | --- | --- | --- | --- | --- | --- | --- |
|  |  | **N = 102** | **Mean OS (months/%^1^)** | **P Value** | **N = 69** | **Mean OS (months/%^1^)** | **P Value** | **N =31** | **Mean OS (months/%^1^)** | **P value** |
| **Total** | | | | | | | | | | |
| **CD68+CD206+** |  |  |  | **0.013** |  |  | **0.041** |  |  | 0.196 |
|  | ≤12.75 | 32 | 71 (57.0) |  | 13 | 72 (56.4) |  | 19 | 60 (57.9) |  |
|  | >12.75 | 70 | 102 (82.2) |  | 43 | 105 (85.5) |  | 27 | 83 (77.4) |  |
| **CD68+iNOS+** |  |  |  | 0.136 |  |  | 0.098 |  |  | 0.458 |
|  | ≤37 | 77 | 95 (76.3) |  | 36 | 106 (84.4) |  | 41 | 74 (68.7) |  |
|  | >37 | 25 | 68 (67.2) |  | 20 | 71 (69.2) |  | 5 | 53 (60.0) |  |
| **CD11b+CD14+**^†^ |  |  |  | **0.044** |  |  | **0.041** |  |  | 0.248 |
|  | ≤3.25 | 33 | 77 (53.2) |  | 20 | 82 (61.9) |  | 13 | 44 (33.8) |  |
|  | >3.25 | 67 | 84 (82.3) |  | 35 | 88 (87.8) |  | 32 | 76 (76.5) |  |
| **CD11b+CD15+**^†^ |  |  |  | **0.050** |  |  | 0.447 |  |  | 0.070 |
|  | ≤3.25 | 51 | 102 (81.1) |  | 31 | 102 (80.6) |  | 20 | 78 (81.8) |  |
|  | >3.25 | 49 | 70 (64.8) |  | 24 | 73 (74.6) |  | 25 | 61 (55.1) |  |
| **CD3+CD4+** |  |  |  | **0.009** |  |  | 0.083 |  |  | **0.040** |
|  | ≤491.2 | 59 | 76 (61.1) |  | 30 | 82 (66.9) |  | 29 | 59 (54.0) |  |
|  | >491.2 | 38 | 104 (89.8) |  | 22 | 109 (90.9) |  | 16 | 86 (88.9) |  |
| **CD3+CD8+** |  |  |  | **<0.001** |  |  | 0.130 |  |  | **0.002** |
|  | ≤91.25 | 10 | 35 (14.6) |  | 1 | 44 (0.0) |  | 9 | 34 (17.8) |  |
|  | >91.25 | 87 | 97 (79.8) |  | 51 | 99 (79.7) |  | 36 | 80 (80.2) |  |
| **CD20+** |  |  |  | **0.005** |  |  | 0.236 |  |  | **0.037** |
|  | ≤53.5 | 10 | 45 (35.0) |  | 2 | 44 (0.0) |  | 8 | 45 (41.7) |  |
|  | >53.5 | 87 | 97 (76.6) |  | 50 | 99 (79.6) |  | 37 | 80 (72.1) |  |
| **Stroma compartment** | | | | | | | | | | |
| **CD68+CD206+** |  |  |  | **<0.001** |  |  | **0.009** |  |  | **0.002** |
|  | ≤2.25 | 16 | 55 (34.9) |  | 11 | 65 (48.5) |  | 5 | 29 (0.0) |  |
|  | >2.25 | 86 | 98 (81.4) |  | 45 | 106 (86.2) |  | 41 | 78 (76.6) |  |
| **CD68+iNOS+** |  |  |  | 0.300 |  |  | 0.400 |  |  | 0.618 |
|  | ≤6.75 | 91 | 93 (75.0) |  | 50 | 100 (80.4) |  | 41 | 73 (67.8) |  |
|  | >6.75 | 11 | 59 (63.6) |  | 6 | 64 (66.7) |  | 5 | 53 (60.0) |  |
| **CD11b+CD14+**^†^ |  |  |  | **0.007** |  |  | **0.013** |  |  | 0.088 |
|  | ≤3.25 | 43 | 77 (55.3) |  | 26 | 82 (62.6) |  | 17 | 43 (43.7) |  |
|  | >3.25 | 57 | 87 (86.5) |  | 29 | 91 (92.5) |  | 28 | 80 (80.1) |  |
| **CD11b+CD15+**^†^ |  |  |  | 0.080 |  |  | 0.283 |  |  | 0.227 |
|  | ≤3.75 | 57 | 100 (79.9) |  | 33 | 102 (82.1) |  | 24 | 73 (76.9) |  |
|  | >3.75 | 43 | 70 (63.8) |  | 22 | 61 (72.2) |  | 21 | 63 (55.1) |  |
| **CD3+CD4+** |  |  |  | **0.011** |  |  | **0.049** |  |  | **0.048** |
|  | ≤247.8 | 49 | 67 (59.3) |  | 28 | 71 (65.1) |  | 21 | 56 (49.5) |  |
|  | >247.8 | 48 | 101 (87.1) |  | 24 | 110 (91.7) |  | 24 | 82 (83.3) |  |
| **CD3+CD8+** |  |  |  | **<0.001** |  |  | 0.130 |  |  | **0.004** |
|  | ≤44 | 11 | 36 (-) |  | 1 | 44 (0.0) |  | 10 | 37 (18.7) |  |
|  | >44 | 86 | 97 (79.7) |  | 51 | 99 (79.7) |  | 35 | 80 (79.8) |  |
| **CD20+** |  |  |  | **0.007** |  |  | 0.093 |  |  | 0.053 |
|  | ≤254.5 | 38 | 59 (55.8) |  | 17 | 66 (59.5) |  | 21 | 53 (52.6) |  |
|  | >254.5 | 59 | 103 (83.0) |  | 35 | 104 (85.5) |  | 24 | 85 (78.8) |  |
| **Tumor compartment** | | | | | | | | | | |
| **CD68+CD206+** |  |  |  | **0.004** |  |  | **0.010** |  |  | 0.162 |
|  | ≤1.25 | 17 | 54 (46.1) |  | 5 | 41 (26.7) |  | 12 | 56(53.6) |  |
|  | >1.25 | 85 | 100 (80.1) |  | 51 | 103 (83.9) |  | 34 | 81 (74.1) |  |
| **CD68+iNOS+** |  |  |  | 0.136 |  |  | 0.098 |  |  | 0.458 |
|  | ≤34.25 | 77 | 95 (76.3) |  | 36 | 106 (84.4) |  | 41 | 74 (68.7) |  |
|  | >34.25 | 25 | 68 (67.2) |  | 20 | 71 (69.2) |  | 5 | 53 (60.0) |  |
| **CD11b+CD14+**^†^ |  |  |  | 0.236 |  |  | 0.167 |  |  | 0.957 |
|  | ≤2.75 | 78 | 88 (70.3) |  | 40 | 93 (72.9) |  | 38 | 72 (67.6) |  |
|  | >2.75 | 22 | 84 (83.3) |  | 15 | 91 (93.3) |  | 7 | 65 (64.3) |  |
| **CD11b+CD15+**^†^ |  |  |  | 0.189 |  |  | 0.212 |  |  | 0.640 |
|  | ≤2.25 | 77 | 88 (69.4) |  | 43 | 94 (74.2) |  | 34 | 71 (64.5) |  |
|  | >2.25 | 23 | 82 (85.9) |  | 12 | 88 (91.7) |  | 11 | 51 (81.8) |  |
| **CD3+CD4+** |  |  |  | **0.007** |  |  | 0.181 |  |  | **0.042** |
|  | ≤46.75 | 20 | 52 (38.3) |  | 5 | 35 (37.5) |  | 15 | 51 (36.7) |  |
|  | >46.75 | 77 | 97 (79.9) |  | 47 | 99 (79.8) |  | 30 | 79 (80.5) |  |
| **CD3+CD8+** |  |  |  | **<0.001** |  |  | 0.130 |  |  | **0.004** |
|  | ≤36.25 | 10 | 40 (14.6) |  | 1 | 44 (0.0) |  | 9 | 40 (17.8) |  |
|  | >36.25 | 87 | 97 (79.8) |  | 51 | 100 (79.7) |  | 36 | 80 (80.2) |  |
| **CD20+** |  |  |  | **<0.001** |  |  | **0.006** |  |  | **0.014** |
|  | ≤14.5 | 11 | 40 (24.2) |  | 4 | 38 (0.0) |  | 7 | 41 (38.1) |  |
|  | >14.5 | 86 | 99 (78.9) |  | 48 | 102 (83.2) |  | 38 | 81 (72.6) |  |

† Abbreviations: CD11b⁺CD15⁺ and CD11b⁺CD14⁺ denote CD11b⁺CD14⁻HLA-DR^low/−^CD15⁺ and CD11b⁺CD14⁺HLA-DR^low/−^CD15⁻, respectively.
